# Urine-Based Metabolomics and Machine Learning Reveals Metabolites Associated with Renal Cell Carcinoma Stage

**DOI:** 10.3390/cancers13246253

**Published:** 2021-12-13

**Authors:** Olatomiwa O. Bifarin, David A. Gaul, Samyukta Sah, Rebecca S. Arnold, Kenneth Ogan, Viraj A. Master, David L. Roberts, Sharon H. Bergquist, John A. Petros, Arthur S. Edison, Facundo M. Fernández

**Affiliations:** 1 Department of Biochemistry and Molecular Biology, University of Georgia, Athens, GA 30602, USA; olatomiwa.bifarin25@uga.edu; 2 Complex Carbohydrate Research Center, University of Georgia, Athens, GA 30602, USA; 3 School of Chemistry and Biochemistry, Georgia Institute of Technology, Atlanta, GA 30332, USA; david.gaul@chemistry.gatech.edu (D.A.G.); ssah9@gatech.edu (S.S.); 4 Department of Urology, Emory University, Atlanta, GA 30308, USA; rsarnol@emory.edu (R.S.A.); kogan@emory.edu (K.O.); vmaster@emory.edu (V.A.M.); jpetros@emory.edu (J.A.P.); 5 Atlanta VA Medical Center, Atlanta, GA 30033, USA; 6 Department of Medicine, School of Medicine, Emory University, Atlanta, GA 30322, USA; drobe04@emory.edu (D.L.R.); shoresh@emory.edu (S.H.B.); 7 Department of Genetics, University of Georgia, Athens, GA 30602, USA; 8 Institute of Bioinformatics, University of Georgia, Athens, GA 30602, USA; 9 Petit Institute of Bioengineering and Bioscience, Georgia Institute of Technology, Atlanta, GA 30332, USA

**Keywords:** renal cell carcinoma, metabolomics, machine learning, liquid chromatography-mass spectrometry, nuclear magnetic resonance spectroscopy, biomarker, tumor metabolism

## Abstract

**Simple Summary:**

Every year, hundreds of thousands of cases of renal carcinoma (RCC) are reported worldwide. Accurate staging of the disease is important for treatment and prognosis purposes; however, contemporary methods such as computerized tomography (CT) and biopsies are expensive and prone to sampling errors, respectively. As such, a non-invasive diagnostic assay for staging would be beneficial. This study aims to investigate urine metabolites as potential biomarkers to stage RCC using machine learning techniques to mine the complex datasets produced. We present a 24-metabolite panel that discriminates between early stage and advanced stage RCC with 87% accuracy in our study cohort.

**Abstract:**

Urine metabolomics profiling has potential for non-invasive RCC staging, in addition to providing metabolic insights into disease progression. In this study, we utilized liquid chromatography-mass spectrometry (LC-MS), nuclear magnetic resonance (NMR), and machine learning (ML) for the discovery of urine metabolites associated with RCC progression. Two machine learning questions were posed in the study: Binary classification into early RCC (stage I and II) and advanced RCC stages (stage III and IV), and RCC tumor size estimation through regression analysis. A total of 82 RCC patients with known tumor size and metabolomic measurements were used for the regression task, and 70 RCC patients with complete tumor-nodes-metastasis (TNM) staging information were used for the classification tasks under ten-fold cross-validation conditions. A voting ensemble regression model consisting of elastic net, ridge, and support vector regressor predicted RCC tumor size with a *R*^2^ value of 0.58. A voting classifier model consisting of random forest, support vector machines, logistic regression, and adaptive boosting yielded an AUC of 0.96 and an accuracy of 87%. Some identified metabolites associated with renal cell carcinoma progression included 4-guanidinobutanoic acid, 7-aminomethyl-7-carbaguanine, 3-hydroxyanthranilic acid, lysyl-glycine, glycine, citrate, and pyruvate. Overall, we identified a urine metabolic phenotype associated with renal cell carcinoma stage, exploring the promise of a urine-based metabolomic assay for staging this disease.

## 1. Introduction

Kidney cancer is one of the deadliest urinary cancers, with an advanced stage (stage III and IV) 5-year survival rate of 12% [1]. In the United States, 76,080 patients are projected to be diagnosed with the disease in 2021, with an estimated death toll of 13,780 [2]. Renal cell carcinomas constitute approximately 90% of kidney and renal pelvis cancers. Because RCC prognosis and treatment depend on accurate staging, innovations in clinical staging are warranted. Currently, preoperative staging is carried out via computerized tomography (CT) scans or MRI, which are expensive and (for CT) expose the patient to ionizing radiation [3]. Non-invasive staging assays using urine samples have the potential of being highly beneficial should they be able to contribute to clinical decision-making. In an earlier study, we applied machine learning and multiplatform metabolomics in urine samples to detect RCC [4]. The study presented here investigates the discrimination of early and advanced RCC stages using advanced machine learning techniques.

It is well established that metabolic reprogramming in cancer contributes to its progression [5,6,7]. As such, changes in metabolite profiles in biofluids such as urine could allow for RCC stage stratification and monitoring. Given the physical proximity of the kidney to urine, the case for a urine-based diagnostic and prognostic indicator for RCC is further strengthened. Mass spectrometry (MS) and Nuclear Magnetic Resonance (NMR) spectroscopy are two popular platforms for metabolomics profiling. In this study, both were combined for maximum coverage of the urine metabolome.

Omics research has been one of the hallmarks of biological research in the 21st century, marked by the rapid growth in the ability to interrogate large datasets by modern statistical techniques, such as machine learning. The metabolomics literature has reflected this technological revolution [8,9,10]. Machine learning (ML) is a subfield of artificial intelligence that involves computer learning of patterns buried in data without being explicitly programmed to do so [11]. This characteristic makes ML a powerful tool for biomarker discovery [9,12].

While many studies have focused on detecting RCC via urine biomarkers, only a small number have investigated biomarkers for RCC staging. In 2020, Liu and co-workers presented a urine metabolic panel for discriminating early and late RCC, using liquid chromatography (LC)-MS. In their study, early RCC consisted of primary tumor stages 1 and 2 (pT1 and pT2), while advanced RCC consisted of primary tumor stages 3 and 4 (pT3 and pT4). The discriminant metabolic panel consisted of thymidine, cholic acid glucuronide, alanyl-proline, isoleucyl-hydroxyproline, and myristic acid [13]. In addition, Falegan et al. showed the potential for discriminating between pT1 and pT3 tumor stages using gas chromatography (GC)-MS of serum samples coupled to Partial Least Squares-Discriminant Analysis (PLS-DA) modeling, but did not identify the chemical structure of the metabolites responsible for such clustering [14]. Furthermore, Monge and co-workers recently reported a 26-lipid panel that discriminates early from late-stage clear cell RCC in human serum samples [15]. Arendowski et al. identified indole-3-acetylglycine, urothion, and myo-inositol 1,4-bisphosphate in urine samples as potential markers to discriminate low stages (pT1 and pT2) from high stages (pT1 and pT2) RCC [16]. Furthermore, Niziol and co-workers, in their urine metabolomics study, identified increasing abundances of isoleucine, N-dimethylglycine, sucrose, and glycolate as RCC tumor grade increases, while urea, 2-fluoroglycine, trigonelline, and 4-hydroxyphenylacetate has the opposite trend [17].

Given the success of our previous metabolomics study in detecting RCC in urine (AUC of 0.98, 88% accuracy) [4], and due to the limited knowledge regarding metabolic pathways useful in RCC stage stratification, we sought to apply advanced ML methods to uncover highly predictive and robust urine biomarkers. Comprehensive tumor, nodes, and metastases (TNM) staging was carried out considering (1) the size of the primary tumor, (2) the presence or absence of metastasis in the regional lymph nodes, and 3) the presence or absence of distant metastasis. In addition, tumor size was predicted using ML through down-selected urinary metabolites. In summary, this study provides evidence that a patient’s urine metabolic profile can be used for accurate RCC stratification.

## 2. Materials and Methods

RCC patients were recruited at Emory University Hospital under an approved IRB protocol. Urine samples were collected at the clinic or at surgery time. Healthy controls were identified during annual physical examinations. All urine samples were collected in sterile tubes and are centrifuged to remove any precipitate and are afterwards stored at −80 °C. Urine samples were thawed on ice and centrifuged to further remove any precipitates before hydrophilic interaction LC-MS and NMR metabolic profiling was conducted, as described previously [4]. For LC-MS experiments, a sample preparation blank and a pooled sample were used for quality control. For NMR experiments, NMR buffer blank, an external control (Nicotine, Ethanol & Drug Free Human Urine, Female; Golden West Diagnostics, LLC) and internal pooled samples were used for quality controls and assurance. Tandem MS was performed for the identification of discriminant features. NMR experiments carried out included one-dimensional nuclear Overhauser effect pulse sequence with pre-saturation of water resonance (NOESYPR1D), two-dimensional (2D) ^1^H-^13^C heteronuclear single quantum correlation (HSQC), and HSQC–TOCSY (HSQC–total correlation spectroscopy). NMR metabolomic features/metabolites are reported with resonances signatures and confidence scores in Table S1. LC-MS data processing conducted using Compound Discoverer V3.0 (ThermoFischer Scientific, Dreieich, Germany). Instrument drift was corrected using a LOESS algorithm. A total 7097 spectral features resulted from the analysis, with 4623 from positive ion mode and 2474 from negative ion mode. For NMR, spectra were aligned using the constrained correlation optimized warping CCOW and normalized using probabilistic quotient normalization (PQN). ML analyses were carried out on the set of combined autoscaled LC-MS and NMR metabolomic features. Complete experimental details are provided in our recent article [4]. 

### 2.1. Tumor Size Estimation

Maximum tumor width was used as a proxy for tumor size to establish which metabolites correlated best with disease stage. Out of the 82 patients in the study with NMR and MS metabolomics measurements, only two had missing tumor sizes. These missing data were replaced with mean imputated values. Pearson’s correlations between metabolites and tumor size were used for metabolomic feature selection, with a cut-off value of 0.55. Elastic net, support vector, ridge, and voting ensemble regression models were used in tumor size predictions. The default parameters in the Scikit-learn library [18] in Python were used for modeling, and 80% and 20% of the data were used for training and testing purposes, respectively.

Ridge regression is a regularized linear model with the goal of minimizing the following objective function during training:

(1) $$R\left(\beta ,\;\lambda \right)=||Y-X\beta |{|}_{2}^{2}+\lambda ||\beta |{|}_{2}{}^{2}$$

where $\beta =\left(\beta_{1}\ldots ,\;\beta_{p}\right){}^{\prime }$ is a vector of slope regression coefficients, $||\cdot |{|}_{2}$ is the $L_{2}$ norm, and λ is a tuning parameter that denotes the regularization strength. λ was set at 1.0. 

Elastic net regression linearly combines the *l*1 and *l*2 regularization of linear model which are lasso and ridge regression respectively. The objective function is as follows:

(2) $$E\left(\beta ,\lambda ,\;\alpha \right)=\frac{1}{2n}||Y-X\beta |{|}_{2}^{2}+\alpha \lambda ||\beta |{|}_{1}+\frac{1}{2}\lambda \left(1-\alpha \right)||\beta |{|}_{2}{}^{2}$$

$\beta ,\;||\cdot |{|}_{2}$**,** and λ are as described in Equation (1). $||\cdot |{|}_{1}$ is the $L_{1}$ norm, and α is the mixing parameter between ridge and lasso regression. λ and α were set to 1.0 and 0.5, respectively.

Support Vector Regressor (SVR) is a nonparametric technique that relies on kernel functions. The objective function of SVR, as opposed to ordinary least square methods, involves minimizing the *l*2 norm of the coefficient vector ($\frac{1}{2}||\beta |{|}^{2}$), and not the squared error term. The objective function is as follows:

(3) $$\frac{1}{2}||\beta |{|}^{2}+C\overset{n}{\underset{i=1}{\sum }}\left|\xi_{i}\right|$$

With the following constraint:

(4) $$\left|y_{i}-\beta_{i}x_{i}\right|\leq \varepsilon +\left|\xi_{i}\right|$$

where β is the coefficient vector, ε is the maximum error—which defines the margin of error acceptable to the model. Additional errors beyond ε are the slack parameters ξ. C is a regularization parameter that accommodates or penalizes ξ. In short, the objective function will be minimized with the constraint that the absolute difference between measured and predicted tumor sizes must be less or equal to the maximum error and absolute slack parameters for samples during training. C and ε were set to 1.0 and 0.1, respectively. The kernel used was a radial basis function.

The voting ensemble regressor is an ensemble of the three regression models above. The base regressors were fit to the dataset, and the average of the output of the individual predictions for each base regressor was computed. All models were evaluated using the coefficient of determination ($R^{2}$), which describes the proportion of variance for the tumor size explained by the urine metabolites predictors. The formula is given below:

(5) $$R^{2}=1-\frac{\sum {\left(y_{i}-{\hat{y}}_{i}\right)}^{2}}{\sum {\left(y_{i}-\bar{y}\right)}^{2}}$$

where $y_{i}$ is the RCC tumor size of patient *i*, ${\hat{y}}_{i}$ is the predicted RCC tumor size of patient *i*, and $\bar{y}$ is the mean RCC tumor size of all patients.

### 2.2. Feature Selection for the RCC Stage Stratification

The normalized abundances of 50 metabolomic features quantified with NMR and >7000 features from LC-MS were combined into one feature table in Python. Features for RCC stratification were retained through the following sequential steps: (1) 1-fold change between the two groups; (2) Student *t*-test with a *p*-value < 0.05; (3) Pearson correlation > 0.8. Before further feature selection, all features were autoscaled. Partial least square discriminant analysis (PLS-DA) was carried out, and the variable importance in projection (VIP) scores were used to select top-ranked features. Similarly, random forest recursive feature elimination (RF-RFE) was conducted, and its Gini Index used to select top-ranked features. Overlapping features from the top 35 ranked features using both PLS-DA and RF-RFE were selected as a metabolite panel for this study.

### 2.3. Machine Learning-Enabled RCC Stage Stratification

In clinical practice, RCC is typically stratified using the tumor, nodes, and metastases (TNM) staging system where tumors are classified into stage I–IV. This staging system was used in this study. The RCC stage stratification was performed by predicting early RCC (stage I and II) and advanced RCC (stage III and IV) with random forest, support vector machine, logistic regression, adaptive boosting, and a voting ensemble classifier. The default parameters in the Scikit-learn library [18] in Python were used for modeling. For training and testing purposes, a 10-fold cross-validation method was applied.

Random forest classification is a collection of decision tree estimators that are constructed with bootstrapped training samples. A decision tree is an inverted tree with a root node, an internal node, and a leaf node. The root and internal nodes are assigned metabolomic features that drive the decisions, while the leaf nodes give the final prediction of either early or advanced RCC. One hundred trees were used in the forest, and the quality of the split was measured by Gini impurity [19].

In support vector machines, the algorithm’s goal is to discover a separating hyperplane, in this case, for a binary classification problem. The decision function takes the following form:

(6) $$\mathrm{RCC}\;\mathrm{score}=\beta_{0}+\sum_{j=1}^{j}\beta_{j}x_{ij}$$

$\beta_{0}$ and $\beta_{j}$ are the bias and the weight parameters of the model, respectively. The index *i* indicates the sample, and *j* represents the metabolomic features. The RCC score determines the class membership. In this formulation, a negative score indicates early RCC, while a positive score indicates advanced RCC, as the separating hyperplane takes the form $\beta_{0}+\beta x^{\prime }=0$. A radial basis function (RBF) kernel was used, and the regulation parameter *C* was set at 1.0.

Logistic regression is an extension of linear regression where predictions are mapped to a class membership via the sigmoid function. The objective function is:

(7) $$\left(\hat{y},y\right)=-\left[ylog\hat{y}+\left(1-y\right)log\left(1-\hat{y}\right)\right]$$

where *y* indicates actual tumor size, and $\hat{y}$ the predicted tumor size. 

Adaptive boosting (AdaBoost) is an ensemble of decision tree classifiers. AdaBoost involves the sequential boosting of its base classifier by ascribing larger weights to misclassified samples to induce the corrections of misclassifications in subsequent decision trees classifier. A linear combination of all base classifiers results in the final decision function. The learning rate was set to 1.0, while the number of decision trees was set to 50. The voting classifier is an ensemble of the four classifiers above. Soft voting was used, where the average probability outputs of the base learners are the voting classifier’s final output.

Binary classifiers were evaluated using the area under the curve (AUC), accuracy, sensitivity, and specificity. AUC is the area under the curve of a receiver operating characteristics (ROC) curve. The ROC curve plots the true positive rate against the false-positive rate, displaying the model’s performance at all classification thresholds. As such, this makes AUC the most desirable metric for binary classification with an unbalanced dataset. AUC was used to select the best models in the study.

Accuracy was calculated as the percentage of all correctly predicted RCC stage samples. (FP is false positive, FN is false negative, TP is true positive, and TN is true negative).

(8) Accuracy= (TP+TN)/(TP+TN+FP+FN)

Sensitivity was calculated as the percentage of correctly-predicted advanced RCC patients out of the total of advanced RCC samples.

(9) Sensitivity= TP/(TP+FN)

Specificity was calculated as the percentage of correctly predicted early-stage RCC patients out of the total of early RCC samples.

(10) Specificity= TN/(TN+FP)

Early RCC is denoted as a negative sample and advanced RCC as a positive sample in this context.

### 2.4. Implementation Environment and Computational Libraries

The Edison Lab’s in-house MATLAB Metabolomics Toolbox (https://github.com/artedison/Edison_Lab_Shared_Metabolomics_UGA, accessed on 11 November 2021, Matlab R2017b, The Mathworks, Inc., Natick, MA, USA) was used for NMR data analysis. Combination of LC-MS and NMR metabolomic features and subsequent computational analysis were carried out using the Python 3.7.0 programming language. Pandas 1.0.5 was used for data handling and manipulations [20]. Matplotlib 3.3.0 and Seaborn 0.10.1 was used in data visualization [21]. NumPy 1.19.1 and SciPy 1.5.1 were used for numerical computing [22,23]. Statsmodel 0.11.1 was the statistical package used [24]. Sci-kit learn 0.23.2, and Yellowbrick 1.3.post1 was used for machine learning [18], and the integrated development environment used was Jupyter notebook [25]. All Jupyter notebooks used in the study can be found in GitHub (https://github.com/artedison/RCC-staging, accessed on 11 November 2021). All datasets are shared in the Metabolomics Workbench (project ID PR001214 and study ID ST001923 and ST001924).

## 3. Results

### 3.1. Patient Cohort Characteristics

**Figure 1:**
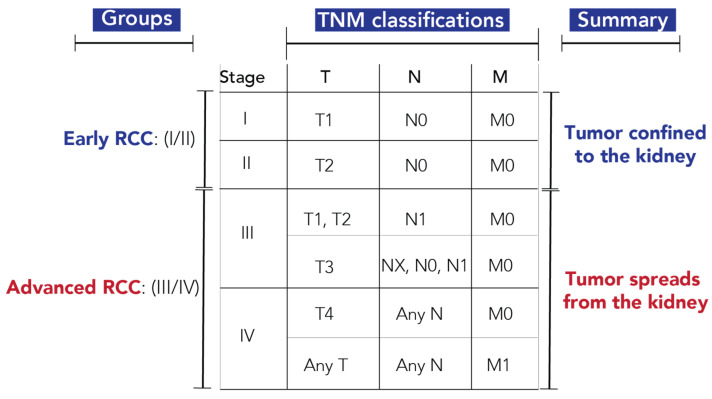
Staging protocol for classification of early- and advanced-stage RCC. Abbreviations: T, primary tumor; T1, the tumor is 7 cm or less in its greatest dimension and limited to the kidney; T2, the tumor is greater than 7 cm in its greatest dimension but limited to the kidney; T3, the tumor extends into major veins or perinephric tissues but not into the ipsilateral adrenal gland and not beyond Gerota fascia; T4, tumor invades beyond Gerota fascia (including contiguous extension into the ipsilateral adrenal gland); N, regional lymph nodes; NX, regional lymph nodes cannot be assessed; N0, no spread to regional lymph nodes; N1, spread to regional lymph node(s); M, distant metastasis; M0, no distant metastasis; M1, distant metastasis [3].

The TNM staging protocol used for tumor stratification is shown in Figure 1 [3]. T indicates the size and extent of the primary tumor, N indicates the presence or absence of tumor spread into the regional lymph nodes, and M indicates the presence or absence of distant metastasis. Stage I and II are classified as early RCC, with the tumor confined to the kidney, while Stage III and IV are classified as advanced RCC, with tumor cells spreading outside of the kidney. Out of the 82 urine samples with metabolomics measurements, twelve samples with inconclusive TNM staging were removed from RCC stratification models. However, all samples were used for tumor size predictions (Table S2).

The relevant clinical and demographics information for the studied cohort are shown in Table 1. We used 41 and 29 urine samples for early and advanced stage RCC, respectively, with no statistically-significant difference between the groups in terms of BMI (*p* = 0.63, Student’s *t*-test) or age (*p* = 0.14, Student’s *t*-test) (Figure S1D,E). The predominant race in both early RCC (*n* = 26, 63.4%) and advanced RCC (*n* = 21, 72.4%) was Caucasian, with a greater proportion of subjects who never smoked in both early RCC (*n* = 24, 58.5%) and advanced RCC (*n* = 19, 65.5%). The proportion of female patients in early RCC was 53.7% (*n* = 21), and 31.1% (*n* = 9) in advanced RCC. To test whether these covariates were potential confounders in the study, principal component analysis (PCA) was applied using the final 24-metabolite panel proposed in the study as features. PCA score plots showed no clustering based on any of the previously named variables (Figure S1A–C). 

### 3.2. Correlation of RCC Tumor Size with Urine Metabolite Abundances

**Figure 2:**
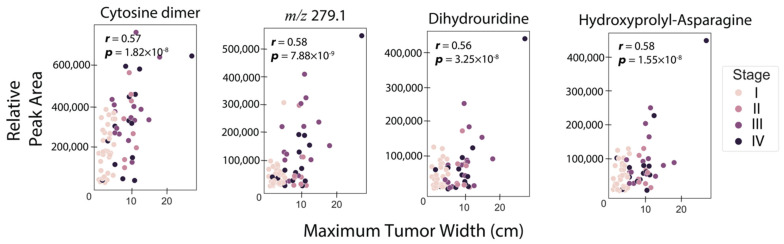
Metabolites with the highest correlation with maximum tumor width. Pearson correlation coefficient and *p*-values for testing non-correlation are provided. The threshold for the correlation coefficient was *r* > 0.55.

An important parameter in RCC is the primary tumor size, which is the original or first tumor. As such, we investigated the strengths of the associations between RCC tumor sizes and individual urine metabolites considered one at a time. To do this, Pearson correlations were computed between tumor size and each metabolite abundance, for both MS and NMR-detected species. Eighty-two samples with associated tumor sizes were included in this analysis, with Table S2 describing the clinical and demographic characteristics of this cohort. Figure 2 shows correlation plots for the four metabolites with the highest associations with tumor size (*r* > 0.55), showing a moderate, but still positive correlation. Three of the four metabolites were annotated as cytosine dimer (*r* = 0.58, *p* = $9.8\times 10^{-8}$), dihydrouridine (*r* = 0.56, *p* = $3.8\times 10^{-8}$), and asparaginyl-hydroxyproline (*r* = 0.57, *p* = $1.8\times 10^{-8}$) (Table 2). Given this moderate positive association of urine metabolites with RCC tumor size, we built ML models to investigate the possibility of estimating tumor size. Elastic net regressor, support vector regressor, ridge regressor, and a voting ensemble regressor combining all three previous models were utilized. The training set consisted of 80% of the data, while the test set was built with the remaining 20%. The best prediction was obtained using the vote regressor with a test set *R*^2^ value of 0.58 (Figure S2a,b), indicating that the model explained 58% of the variation in tumor size. The results for other models such as the elastic net regressor (train *R*^2^ = 0.46, test set *R*^2^ = 0.56), support vector regressor (train *R*^2^ = 0.23, test set *R*^2^ = 0.51), and ridge regressor model (train *R*^2^ = 0.46, test set *R*^2^ = 0.56), are shown in Figure S2c–h, with an explained tumor size variation range of 51–56% in the test set.

### 3.3. Machine Learning Accurately Discriminates Early and Advanced Stage RCC

NMR and LC-MS metabolic features were combined into a single table, and 16 discriminating metabolites were selected using the pipeline described in Figure S3. First, two filter-based approaches were used for feature selection: (1) metabolic features with greater than 1-fold difference between the early and advanced RCC, followed by (2) a Student *t*-test between the two groups (cut off value, *p* < 0.05). This resulted in a subset of 171 LC-MS metabolic features (Table S3). Then, a Pearson correlation-based method was used with a cut-off value of *r* > 0.8 to remove highly correlated features that might degrade machine learning predictions. This procedure left 99 metabolic features remaining in the feature table. Finally, embedded feature selection techniques were layered inside a voting-based system for the final biomarker selection. A partial least squares regression technique was used to rank feature importance via their Variable Importance in Projection (VIP) scores, and the top 35 metabolic features were selected. In addition, a random forest recursive feature elimination technique was used to rank feature importance via its Gini index, and the top 35 features were also selected. As a voting system, features that appeared on both lists were selected after the removal of those that did not. This led to a 16-urine metabolite panel for RCC stage stratification (Table 3). Identified metabolites included 4-guanidinobutanoic acid, 7-aminomethyl-7-carbaguanine, N-alpha-N-alpha-dimethyl-L-histidine, diethyl-2-methyl-3-oxosuccinate, 3-hydroxyanthranilic acid, apo-[3-methylcrotonoyl-CoA:carbon-dioxide ligase (ADP-forming)], lys-gly/gly-lys, and succinic anhydride. All these markers were detected by LC-MS. Random forest (RF), adaptive boosting (AdaBoost), support vector machine with radial basis function kernel (SVM-RBF), logistic regression, and a voting ensemble combining all four methods were used for stratification under ten-fold cross-validation conditions. Figure S4 shows the ML predictions for RCC staging using the 16-metabolite panel and various classifiers. Overall, voting ensemble models gave the best predictions, with an AUC of 0.95, accuracy of 86%, sensitivity of 80%, and specificity of 91%.

Because NMR metabolomic features were underrepresented in the initial dataset when compared to MS, and therefore less likely to be selected in the metabolite panel, we added NMR metabolomic features with a *p*-value lower than 0.05 (Student’s *t*-test) to this panel to examine if this strategy could further improve predictions. The metabolites included were citrate, glycine, choline, acetone, and pyruvate as shown in Table 4.

**Figure 3:**
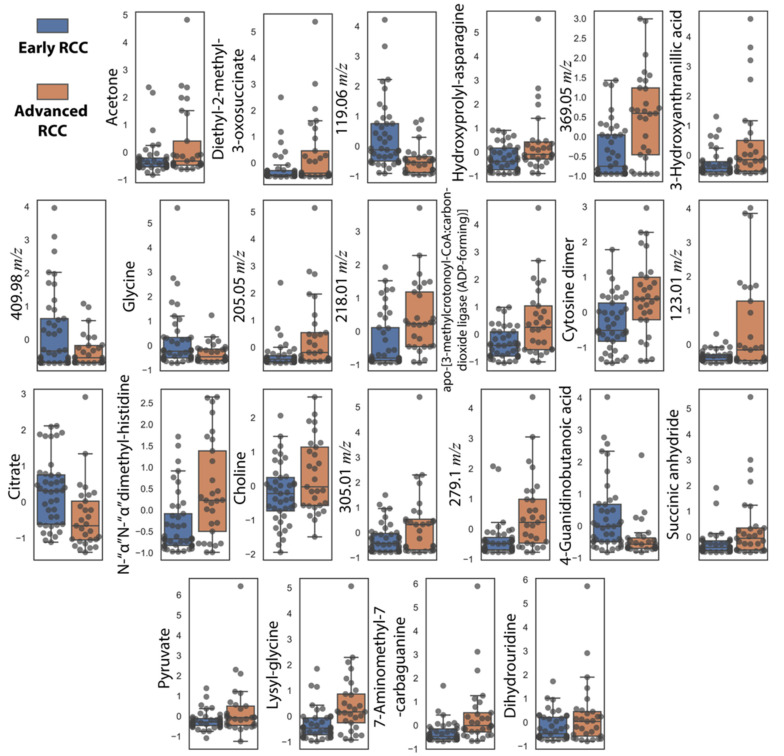
Box plots showing autoscaled normalized relative abundances for the 24 metabolite-panel to distinguish early-stage RCC (*n* = 41) from advanced-stage RCC (*n* = 29). The mean, upper quartile, lower quartile, minimum, and maximum values are shown. All metabolites had *p*-values < 0.05 (Student *t*-test).

**Figure 4:**
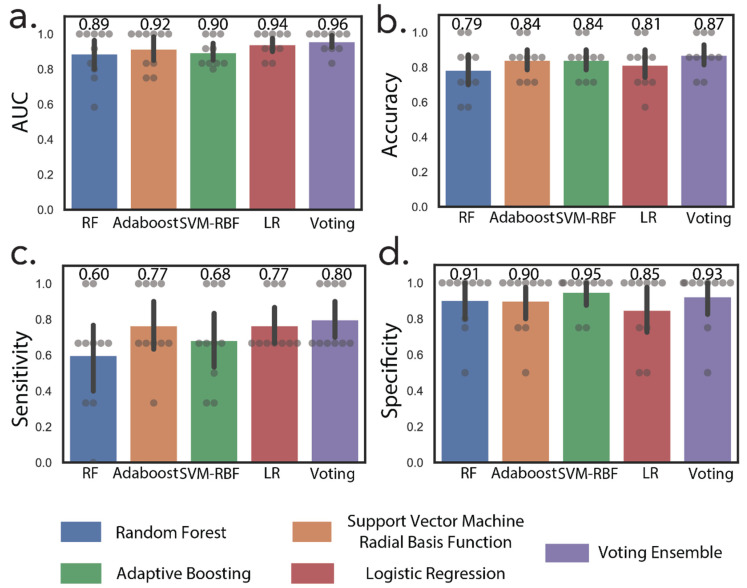
Machine learning discriminates between early-stage and advanced-stage RCC. Machine learning predictions by random forest, AdaBoost, support vector machine radial basis function (SVM-RBF), logistic regression (LR), and voting ensembles using the 24-metabolite panel. (**a**) Area under the ROC curve, (**b**) accuracy, (**c**) sensitivity, (**d**) specificity.

In addition to NMR metabolites, we also included metabolites correlated with increasing tumor size that were missing in the 16-metabolite panel. These include cytosine dimer, dihydrouridine, and hydroxyprolyl-asparagine (Table 2). These subsequent additions resulted in a 24-metabolite panel, which was considered as the final panel. Normalized relative urine abundances for metabolites in this panel are shown in Figure 3. As for the previous panel, random forest, AdaBoost, SVM-RBF, logistic regression, and a voting ensemble combining all four methods were used for stratification under ten-fold cross-validation conditions. Figure 4 shows ML predictions of RCC stage using the 24-metabolite panel. Again, the voting ensemble classifier gave the best predictions with an AUC of 0.96, a slightly higher classification score than for the 16-metabolite panel. Other prediction scores included 87% accuracy, 80% sensitivity, and 93% specificity. In addition, the prediction results for each metabolite in the panel are presented in Table S4.

### 3.4. Comparison of RCC Stages and Healthy Controls Reveals Metabolic Trends of RCC Staging Markers

The relevant demographics and clinical information for the healthy control cohort are shown in Table S5. None of the markers from our previous RCC detection study were selected as RCC staging marker presented here [4]. However, 13 of the 24 biomarkers in the staging panel have *p*-values less than 0.05 (Welch’s *t*-test) when all RCC samples (*n* = 82) were compared with the healthy controls (*n* = 174) (Table S6). Of those 13 metabolites, glycine, citrate, and an unidentified metabolite had interesting trends. While the relative abundances of glycine and citrate decreased in advanced RCC vs. early RCC, their abundances increased in RCC compared to healthy controls. Furthermore, 55 metabolic features were identified as significant (Welch’s *t*-test, BH *q* < 0.05; Log2 FC > 2) when healthy controls (*n* = 174) were compared with early-stage RCC (*n =* 41) (Table S7). While none of the RCC staging markers were present in this set, 13 of the 24 staging markers had *p*-values less than 0.05 (Welch’s *t*-test) when early RCC (*n* = 41) was compared with healthy controls (*n* = 174) (Table S8). In addition, 52 metabolic features were identified as statistically significant (Welch’s *t*-test, BH *q* < 0.05; Log2 FC > 2) when healthy controls (*n* = 174) were compared with advanced stage RCC (*n =* 29) (Table S9). Of these metabolites, 7-aminomethyl-7-carbaguanine was the only one in the RCC staging panel. Furthermore, 14 of the RCC stage biomarkers had *p*-values less than 0.05 (Welch’s *t*-test) (Table S10). Table 5 summarizes the trend of the RCC staging markers in the two RCC stages comparison with healthy controls.

## 4. Discussion

Monitoring tumor progression through specific biofluid metabolite profiles presents a significant translational opportunity. One known characteristic of tumor progression is metabolic rewiring [26]. Comparison of normal and tumor tissues has revealed significant dysregulation in nucleotide biosynthesis, oxidative phosphorylation, glycolysis, and pentose phosphate pathway, amongst others [26]. Results presented in this study confirm those findings, suggesting the capability to identify RCC stage via urine-based metabolomics. The combined use of LC-MS and NMR for metabolic profiling together with machine learning for mining the dataset and identifying the metabolic features that best discriminates early-stage and advanced-stage RCC yielded a twenty-four-metabolite panel that successfully enabled RCC staging.

Examination of the aforementioned panel revealed evidence of upregulated nucleotide metabolism with an increase in the abundances of cytosine dimer, 7-aminomethyl-7-carbaguanine, and dihydrouridine (DHU) in advanced stage RCC urine samples. Increased nucleotide metabolism is a hallmark of tumorigenesis, as it directly supports uncontrolled cell growth [27] via the pentose phosphate pathway [28]. These increased abundances also explain the correlation of urinary cytosine dimer and DHU with RCC tumor size. Cytosine is present in both DNA and RNA, while DHU is found in tRNA as a nucleoside. Together, these metabolites suggest increased nucleotide metabolism in RCC [29]. An additional pyrimidine metabolite in the panel, 7-aminomethyl-7-carbaguanine, is one of the precursors for the synthesis of queuosine, a modified analogue of guanosine found in the first anticodon loop of tRNAs for histidine, aspartic acid, asparagine, and tyrosine [30]. This tRNA modification has been reported to promote cell proliferation in cancer in mouse models [31]. In addition, queuine tRNA-ribosyltransferase (QTRT1), the enzyme that catalyzes the hypermodification of queuosine using 7-aminomethyl-7-carbaguanine, is highly expressed in lung adenocarcinoma (LUAD) [32], and has been identified as a risk factor for the progression of LUAD [32]. This trend has also been reported in other human malignant tumors [33,34], and breast cancer cell lines [35].

The relative abundance of 3-hydroxyanthranilic acid was increased in advanced RCC patients’ urine samples. This metabolite is an intermediate of tryptophan metabolism, a metabolic pathway that has been also implicated in a recent RCC urine metabolomics study [13]. In that study, N-formylkynurenine, a metabolite upstream of hydroxyanthranilic acid, was selected as a putative marker that discriminated malignant RCC tumors from both healthy cohort and benign RCC tumors. In fact, studies from as early as 1975 have reported higher levels of 3-hydroxyanthranilic acid in untreated bladder and kidney carcinoma patients [36]. Indeed, 3-hydroxyanthranilic acid has been shown to promote tumor immune evasion [37,38,39].

Two dipeptides, hydroxyprolyl-asparagine, and lysyl-glycine had elevated levels in advanced RCC urine samples in our study. Numerous dipeptides had been reported to increase at advanced RCC stages (III and IV) in a paired clear cell renal cell carcinoma (ccRCC)/normal tissue study [40]. The presence of these increased dipeptide levels might be indicative of increased protein degradation and reutilization processes [41,42]. In addition, a previous urine metabolomics study reported the dipeptides alanyl-proline and isoleucyl-hydroxyproline as being elevated in RCC pT3 and pT4 stages [13].

Lower levels of guanidinobutanoic acid, a gamma-amino acid and uremia toxin, were found in advanced stage RCC. This might be due to the progressive retention of the metabolite that is otherwise excreted normally in healthy kidneys [43]. Apo-[3-methylcrotonoyl-CoA:carbon-dioxide ligase (ADP-forming)] is involved in the biotin metabolism pathway, indicating a possible alteration in biotin metabolism in advanced RCC. Likewise, diethyl-2-methyl-3-oxosuccinate and N,N-dimethyl-histidine might indicate alterations in succinate and histidine metabolism, respectively; while succinic anhydride is likely an exogenous metabolite that is used in food additives [44].

NMR-derived metabolites in the panel included citrate, glycine, choline, acetone, and pyruvate. Reduced levels of citrate and increased levels of pyruvate suggest a dysregulated aerobic glycolytic pathway in RCC [45]. This dysregulation is required to maintain cell proliferation processes that characterize tumors, and therefore differences in metabolite abundances in this pathway are expected as the tumor progresses. The abundance of citrate has been reported to decrease in urine metabolomics studies comparing healthy controls or benign with malignant RCC tumors [46,47]. Citrate has also been linked to drive increased fatty acid synthesis in tumors [48], and the overexpression of ATP citrate lyase has been reported as RCC progresses [49]. ATP citrate lyase links carbohydrate metabolism to fatty acid biosynthesis via the conversion of citrate to acetyl-CoA. In addition, elevated pyruvate levels, another evidence of dysregulated glucose metabolism, were reported in a urine metabolomics study that compared benign RCC tumors with malignant RCC [47]. The lower levels of glycine abundance in advanced RCC urine samples agree with the role of glycine in rapid cancer cell proliferation [50,51,52,53]. In a study that used MS to measure the consumption and release of metabolites in media of NCI-60 cancer cell lines, glycine consumption and the expression of mitochondrial glycine biosynthetic pathway correlated with proliferation of cancer cells [50]. This observation is linked to the fact that glycine can contribute to both purine and pyrimidine biosynthesis [51,52], therefore playing a pivotal role in sustaining cancer cell growth [53]. Indeed, urinary glycine has been shown to decrease in response to RCC cancer development when benign tumors are compared to malignant tumors [14]. The higher relative abundance of choline in advanced RCC might be caused by increased levels of choline-containing compounds that have been reported in tumors [54]. These compounds are a major component of cell membranes required for cell proliferation [54]. Magnetic resonance spectroscopic imaging has been used to show that total choline is associated with the aggressiveness of breast cancer [55] and prostate cancer [56] and could therefore be also associated with RCC. In addition, it has also been used in the detection and grading of brain tumors [57,58]. The increased levels of acetone in advanced RCC can be explained in light of the increase in the level of ketone bodies associated with some cancers [59]. Indeed, an increase in the level of acetoacetate, a ketone detected by NMR, was observed in our study. This higher abundance of ketone bodies might have been caused by the well-known Warburg effect, which leads to an accumulation of acetyl-CoA and, in turn, increased production of ketone bodies [59]. In an in vitro metabolomics study, ketones were found to increase significantly in the exometabolome of RCC cells compared to a non-tumor cell line [60].

Comparison of early RCC and advanced RCC groups with healthy control samples led to additional insights on the trends of the RCC staging markers. Apo-[3-methylcrotonoyl-CoA:carbon-dioxide ligase (ADP-forming)], dihydrouridine, acetone, pyruvate, hydroxyprolyl-asparagine, 7-aminomethyl-7-carbaguanine, and lys-gly/gly-lys, all increased when both RCC stages were compared to healthy controls, and they also increased when advanced RCC samples were compared to early RCC samples indicating that these metabolites are potential candidates for discriminating advanced RCC from early RCC, and both RCC stages from healthy controls. In addition, the abundances of N,N-dimethyl-histidine, succinic anhydride, diethyl-2-methyl-3-oxosuccinate, cytosine dimer, 3-hydroxyanthranilic acid, and choline are higher in healthy controls when compared to early RCC. However, they increase in advanced RCC when compared to control samples indicating a distinctive increased abundance in advanced RCC. Furthermore, as discussed above, the relative abundances of glycine and citrate decreased in advanced RCC vs. early RCC. However, both metabolites have higher relative abundances in early RCC when early RCC samples were compared to healthy controls compared to their abundances in advanced RCC vs. healthy controls. This trend underscores the argument that these metabolites are clearly linked to tumor proliferation [48,50]. Finally, 4-guanidinobutanoic acid increased in early RCC compared to healthy controls. On the other hand, the metabolite decreases in advanced RCC vs. healthy controls, which agrees with the hypothesis of the progressive retention of the metabolite in the kidney as RCC progresses [43].

## 5. Conclusions

Overall, our study reveals metabolites and pathway alterations associated with RCC stage, with a panel of metabolites accurately discriminating between early RCC and advanced RCC. Furthermore, comparing RCC stages groups with healthy controls reveals interesting RCC marker trends. Despite these, there are a few limitations to point out in this study, (1) group samples sizes are relatively small, and (2) our cohort is not diverse enough, and tests sets did not employ independent samples. However, these encouraging results prompt us to seek further validation of these biomarkers, especially in larger and independent cohorts in future research efforts. In addition, given that some of the metabolite changes in this study are reported in other cancers, this also necessitates the need for further studies into multi-organ cancer metabolomic comparisons.

## Figures and Tables

**Table 1:** Patient cohort characteristics.

	Early RCC	Advanced RCC
No of Urine Samples	41	29
Mean Age ± SD	60.1 ± 13.3	61.6 ± 13.2 ^a^
Mean BMI ± SD	29.9 ± 5.2	27.9 ± 6.2 ^b^
Race		
Caucasian	26 (63.4%)	21 (72.4%)
Black/African American	14 (34.1%)	5 (17.2%)
American-Indian/Alaskan- Native	1 (2.4%)	1 (3.4%)
Mixed	-	1 (3.4%)
Unknown/Missing	-	1 (3.4%)
Smoker		
Never	24 (58.5%)	19 (65.5%)
Former/Current	17 (41.5%)	10 (34.5%)
Gender		
Male	19 (46.3%)	20 (68.9%)
Female	22 (53.7%)	9 (31.1%)
Histological Subtypes		
Pure Clear Cell	23 (56.1%)	26 (89.6%)
Papillary	9 (21.9%)	1 (3.4%)
Clear Cell Papillary	4 (9.8%)	-
Chromophobe	4 (9.8%)	-
Unclassified	1 (2.4%)	2 (6.9%)
Nuclear Grade		
1	-	-
2	21 (51.2%)	3 (10.3%)
3	17 (41.5%)	10 (34.5%)
4	3 (7.3%)	16 (55.2%)
RCC Stage		
I	33 (80.5%)	-
II	8 (19.5%)	-
III	-	15 (51.7%)
IV	-	14 (48.3%)

*p*-values were calculated using the Student *t*-test. ^a^ Age *p*-value = 0.63, ^b^ BMI *p*-value = 0.14. Twelve samples with missing TNM staging information were excluded.

**Table 2:** Compound annotation for the metabolites with the highest correlation (*r* > 0.55) with RCC tumor size.

ID No.	Retention Time (min)	*m*/*z*		Adduct Type	Mass Error (ppm)	Elemental Formula	Metabolite Name	Confidence Level
		Theoretical	Experimental					
2745	1.87	223.0938	223.0936	[M + H]^+^	−0.64	C_8_H_10_N_6_O_2_	cytosine dimer	2
3163	3.53	279.1187	279.1194	[M + H]^+^	2.54	C_10_H_18_N_2_O_7_	-	4
5362	3.46	245.0774	245.0775	*[M − H]^−^*	0.61	C_9_H_14_N_2_O_6_	dihydrouridine	2
6681	2.80	244.0933	244.0934	[M − H]^−^	0.31	C_9_H_15_N_3_O_5_	hydroxyprolyl-asparagine/asparaginylhydroxyproline	2

Metabolite identification level was assigned based on the following criteria: (1) exact mass, isotopic pattern, retention time, and MS/MS spectrum of chemical standard matched to the feature; (2) exact mass, isotopic pattern, and MS/MS spectrum matched with literature spectra or fragmentation ions observed are consistent with the proposed structure; (3) tentative ID assignment based on elemental formula match with literature; (4) unknowns.

**Table 3:** Compound annotation for the 16-metabolite panel (*m*/*z* = mass-to-charge ratio, min = minutes, ppm = part per million).

ID	Retention Time (min)	*m*/*z*		Adduct Type	Mass Error (ppm)	Elemental Formula	Metabolite Identity	Confidence Level
		Theoretical	Experimental					
1372	3.94	146.0924	146.0924	[M + H]^+^	0.03	C_5_H_11_N_3_O_2_	4-guanidinobutanoic acid	2
1904	4.00	180.0879	180.0880	[M + H]^+^	0.08	C_7_H_9_N_5_O	7-aminomethyl-7-carbaguanine	2
2122	1.20	184.1081	184.1080	[M + H]^+^	−0.36	C_8_H_13_N_3_O_2_	N,N-dimethyl-histidine	2
2317	0.89, 0.89	203.0913, 422.2020	203.0912, 422.2023	[M + H]^+^	−0.44 0.71	C_9_H_14_O_5_	diethyl-2-methyl-3-oxosuccinate	3
2465	0.89, 0.89	154.0498 136.0393	154.0497, 136.0392	[M + H]^+^	−0.62 −0.73	C_7_H_7_NO_3_	3-hydroxyanthranilic acid	2
3163	3.53	279.1187	279.1194	[M + H]^+^	2.54	C_10_H_18_N_2_O_7_	--	4
3766	3.63	174.1237	174.1238	[M + H]^+^	0.37	C_7_H_15_N_3_O_2_	apo-[3-methylcrotonoyl-CoA:carbon-dioxide ligase (ADP-forming)]	2
4116	3.79	119.0577	119.0580	[M + H]^+^	4.51	C_4_H_8_NO_3_	--	4
5045	3.49	218.0129	218.0123	[M − H]^−^	−3.50	C_7_H_9_NO_5_S	--	4
5420	3.38	205.0526	205.0535	[M − H]^−^	4.32	C_4_H_12_N_6_P_2_	--	4
5437	0.76	123.0114	123.0108	[M − H]^−^	−4.47	C_9_H_2_N	--	4
5713	1.23	305.0990	305.0989	[M − H]^−^	−0.58	C_11_H_18_N_2_O_8_	--	4
5737	3.99	202.1197	202.1190	[M − H]^−^	−3.58	C_8_H_17_N_3_O_3_	lys-gly/gly-lys	2
5985	0.94	99.0087	99.0088	[M − H]^−^	0.21	C_4_H_4_O_3_	succinic anhydride	2
6687	0.86	369.0517	369.0502	[M − H]^−^	−4.30	C_6_H_14_N_10_O_5_S_2_	--	4
6694	3.82	409.9786	409.9770	[M − H]^−^	−3.47	C_4_H_12_N_7_O_10_P_3_	--	4

Metabolite identification level was assigned based on the following criteria: (1) exact mass, isotopic pattern, retention time, and MS/MS spectrum of standard matched to the feature; (2) exact mass, isotopic pattern, and MS/MS spectrum matched with literature spectra or fragmentation ions observed are consistent with the proposed structure; (3) tentative ID assignment based on elemental formula match with literature; (4) unknowns.

**Table 4:** Annotated NMR metabolites with a *p*-value less than 0.05. These were added to the 16-metabolite panel.

Metabolite/Features	^1^H (ppm)	^13^C(ppm)	Peak Patterns	Confidence Score	Fold Change	*p*-Value
acetone	2.23	32.40	(s)	3	0.49	0.029
pyruvate	2.41	-	(s)	2	0.31	0.028
citrate	2.53	48.52	(d)	3	−0.54	0.003
choline	3.19	56.69	(s)	3	0.22	0.026
glycine	3.56	44.18	(s)	3	−0.66	0.032

s = singlet, d = doublet. Fold change (FC) was calculated as the base 2 logarithm of the mean integral ratios between advanced RCC and early RCC samples. Positive FC values indicate increased abundance in advanced RCC, while negative values indicate higher abundance in early RCC. *p*-values were Student’s *t*-test. Confidence score: (1) putatively characterized compound classes, or annotated compounds, (2) matches from 1D NMR to literature and/or 1D BBiorefcode compound (AssureNMR) or other database libraries such as Biological Magnetic Resonance Bank (BMRB) and Human Metabolome Database (HMDB) (3) matched to Heteronuclear Single Quantum Coherence (HSQC).

**Table 5:** Comparison of early-stage and advanced-stage RCC with healthy controls using the RCC staging markers. Fold change (FC) was calculated as the base two logarithm of the average intensity ratios between two groups.

Metabolite or ID	Early RCC/Healthy Controls	Advanced RCC/Healthy Controls	Advanced RCC/Early RCC
citrate	0.39	−0.16	−0.54
choline	−0.21	0.02	0.22
glycine	0.82	0.16	−0.66
3-hydroxyanthranilic acid	−0.87	0.53	1.41
5045	−1.05	−0.02	1.03
cytosine dimer	−0.41	0.29	0.70
lys-gly/gly-lys	0.73	1.87	1.14
7-aminomethyl-7-carbaguanine	0.69	2.07	1.38
5713	−0.49	0.53	1.01
hydroxyprolyl-asparagine/asparaginylhydroxyproline	0.50	1.44	0.93
pyruvate	0.09	0.40	0.31
4-guanidinobutanoic acid	0.49	−0.63	−1.12
diethyl-2-methyl-3-oxosuccinate	−0.82	0.69	1.51
succinic anhydride	−0.50	1.03	1.53
acetone	0.16	0.65	0.49
3163	−0.36	1.17	1.53
N,N-dimethyl-histidine	−0.24	0.87	1.12
dihydrouridine	0.22	1.07	0.80
5420	0.22	1.95	1.73
4116	−0.09	−1.33	−1.24
apo-[3-methylcrotonoyl-CoA:carbon-dioxide ligase (ADP-forming)]	0.01	1.05	1.04
6687	−2.53	−1.20	1.33
5437	−1.67	0.50	2.18
6694	−1.02	−2.32	−1.30

Positive FC values indicate increased abundance in the first group (numerator), while negative values indicate higher abundance in the second group (denominator).

## Data Availability

The datasets collected in this work are available through the NIH Metabolomics Workbench [61] with the project ID of PR001214 and study IDs ST001923 and ST001924. The dataset can be accessed via http://dx.doi.org/10.21228/M8SH7V, accessed on 11 November 2021.

## Supplementary Materials

The following are available online at https://www.mdpi.com/article/10.3390/cancers13246253/s1, Figure S1. Potential confounder analysis for RCC stage stratification; Figure S2. RCC primary tumor size predictions; Figure S3. Machine learning pipeline for the biomarker selection for RCC stage stratification; Figure S4. Machine learning predictions for RCC stratification using the 16-metabolic panel; Table S1. NMR Metabolomic features; Table S2. RCC patient cohort characteristics for the 82 subjects used for tumor size predictions; Table S3. MS metabolomic features used in RCC stage stratification with *p*-values < 0.05 and >1-fold change; Table S4. Machine learning prediction results for individual metabolites in the RCC staging metabolomic panel; Table S5. Demographics/clinical information for the healthy control cohort; Table S6. RCC staging markers that are statistically significant when RCC samples were compared with the healthy controls; Table S7. Metabolomic features identified as statistically significant when healthy controls were compared with early stage RCC; Table S8. Comparison of RCC staging markers in early staged RCC vs. healthy controls; Table S9. Metabolomic features identified as statistically significant when healthy controls were compared with advanced stage RCC; Table S10. Comparison of RCC staging markers in advanced staged RCC vs. healthy controls.
