# Supplementary material for: Urine-Based Metabolomics and Machine Learning Reveals Metabolites Associated with Renal Cell Carcinoma Stage
Source: Cancers (Basel). 2021 Dec 13;13(24):6253. doi: 10.3390/cancers13246253 (PMC8699523; doi:10.3390/cancers13246253)
Supplement: Supplementary file 1 [file cancers-13-06253-s001.zip › cancers-1482390-supplementary.pdf]

cancers

MDPI

Supplementary Information

Urine-based Metabolomics and Machine Learning Reveals Metabolites Associated with Renal Cell Carcinoma Stage

Olatomiwa O. Bifarin, David A. Gaul, Samyukta Sah, Rebecca S. Arnold, Kenneth Ogan, Viraj A Master, David L. Roberts, Sharon H. Bergquist, John A. Petros, Arthur S. Edison and Facundo M. Fernández.

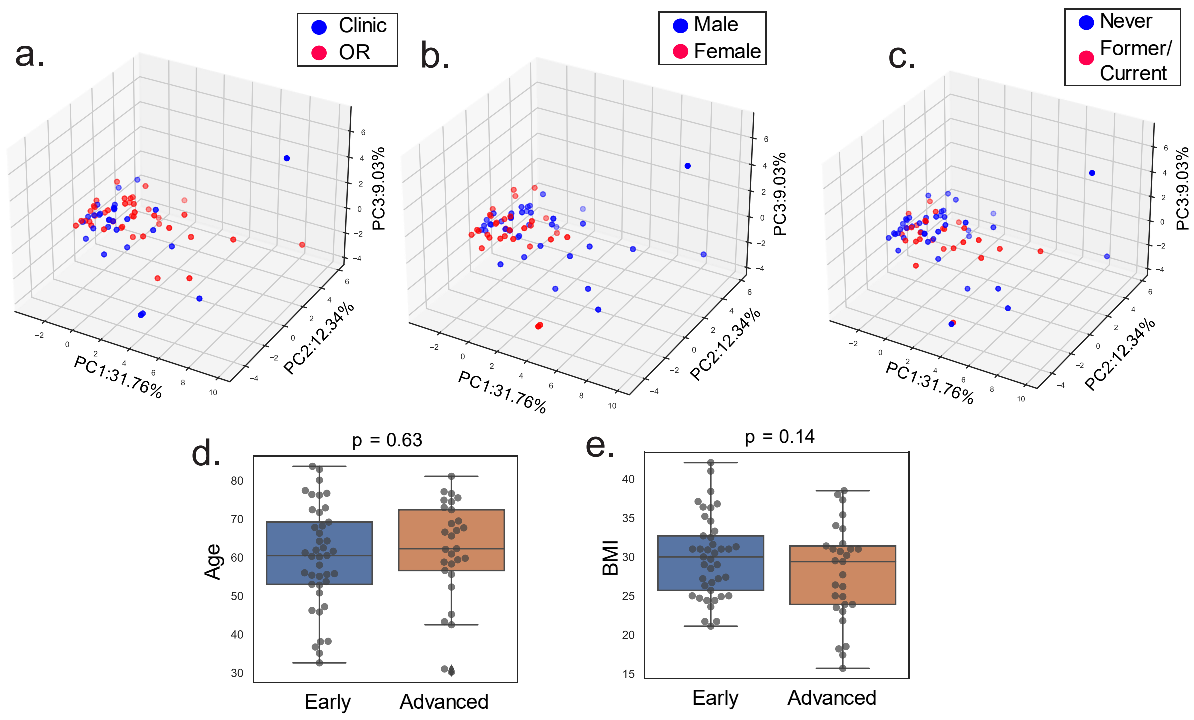

Figure S1. Potential confounder analysis for RCC stage stratification. PCA was conducted using the 24-metabolite panel as features. PCA shows that collection method (a), gender (b), and smoking history (c) are not discriminated by the selected biomarker panel. Age (d) and BMI (e) in the cohort are statistically insignificant between early and advanced RCC patients (Student's *t*-Test).

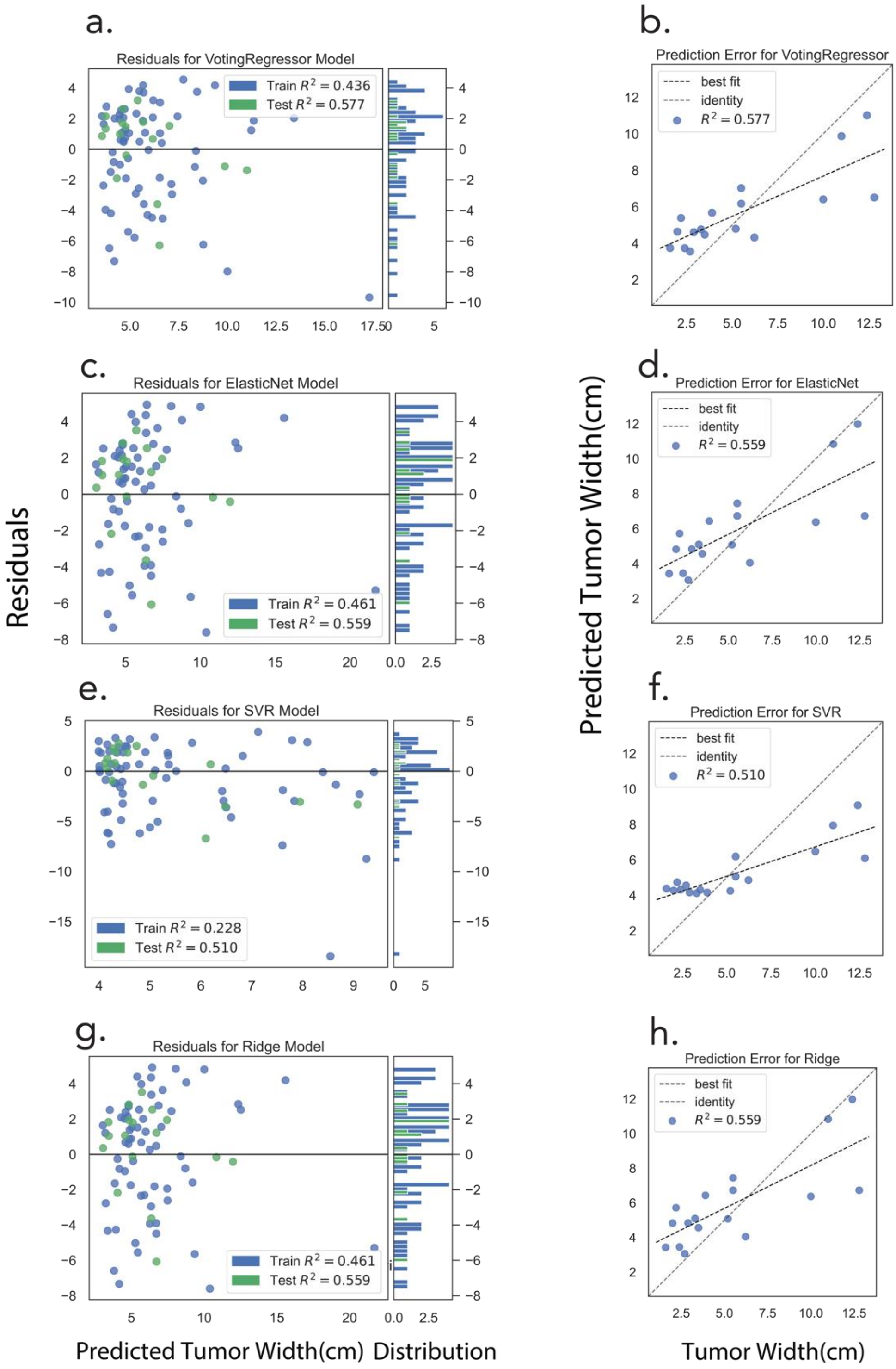

Figure S2. RCC primary tumor size estimation. **(a)** Voting regression model residual plot. **(b)** Voting regression model prediction error plot. **(c)** Elastic net regression model residual plot **(d)** Elastic net regression model prediction error plot. **(e)** Support vector regression model residual plot. **(f)** Support vector regression model prediction error plot. **(g)** Ridge model residual plot. **(h)** Ridge model

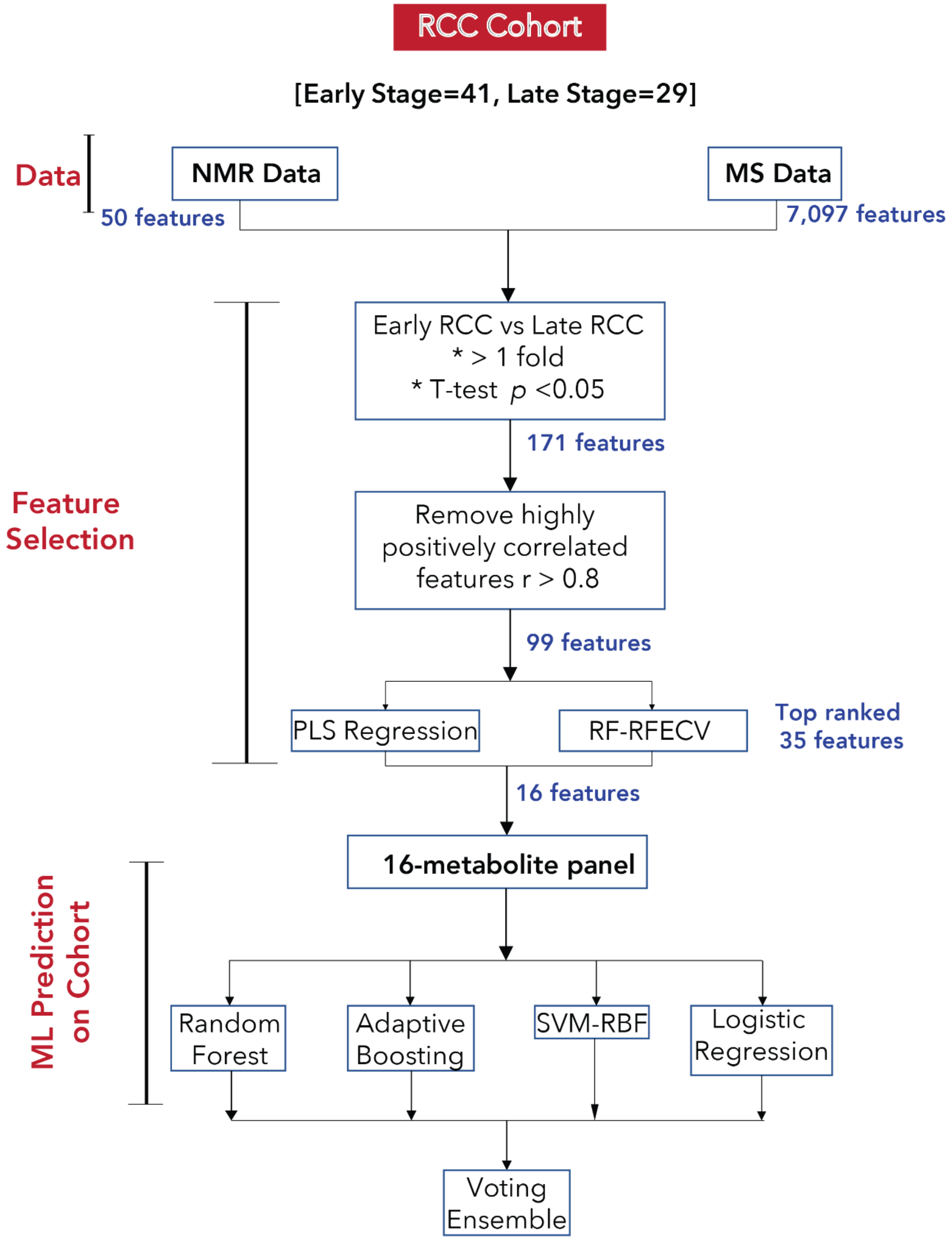

Figure S3. Machine learning pipeline for selection of biomarkers for RCC stage stratification. All NMR and MS features were subjected to a hybrid method of feature selection resulting in a 16-metabolite panel. Machine learning predictions were carried out by four different algorithms and a voting ensemble. PLS: partial least squares. RF- RFECV: random forest recursive feature elimination – cross validation. SVM-RBF: support vector machines radial basis function.

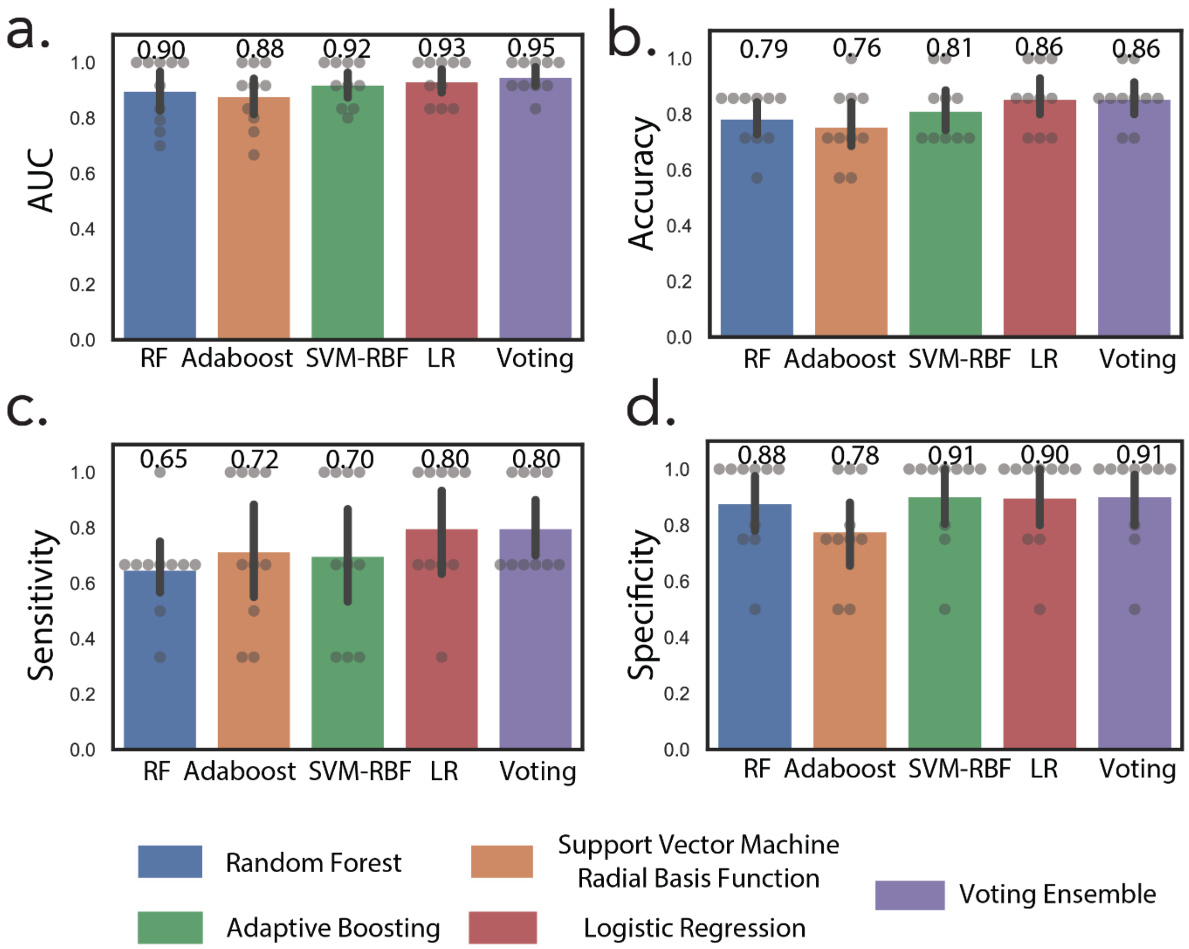

Figure S4. Machine learning predictions for RCC stage stratification using the 16-metabolic panel. a) Area under the ROC curve, (b) accuracy, (c) sensitivity, (d) specificity.

Table S1. NMR-detected metabolites.

Metabolite/Features	^{1}H (ppm)	^{13}C (ppm)	Peak patterns	Confidence Score	Fold Change	*p*-value	*q*-value
unknown 1	0.15	-	(s)	-	0.13	0.604	0.829
unknown 2	0.36	-	(m)	-	-0.1	0.483	0.724
[**]bile acid 1	0.53	-	(s)	1	-0.11	0.473	0.724
[**]bile acid 2	0.56	-	(s)	1	0.04	0.78	0.868
3-hydroxyisovaleric acid	1.26	30.84	(s)	3	-0.17	0.213	0.554
lactate	1.31	22.97	(d)	4	0.18	0.426	0.724
unknown 3	1.85	-	(s)	-	0.63	0.277	0.554
acetate	1.90	26.04	(s)	3	-0.22	0.456	0.724
acetone	2.23	32.40	(s)	3	0.49	0.029	0.306
unknown 4	2.26	-	(s)	-	-0.16	0.202	0.554
acetoacetate	2.27	32.19	(s)	3	0.96	0.056	0.404
unknown 5	2.33	-	(s)	-	-0.03	0.842	0.868
¨pyruvate	2.41	-	(s)	2	0.31	0.028	0.306
citrate	2.53	48.52	(d)	3	-0.54	0.003	0.122
dimethylamine (DMA)	2.71	37.5	(s)	3	0	0.989	0.989
unknown 6	2.77	-	(s)	-	-0.2	0.141	0.554
methylguanidine	2.82	30.21	(s)	3	-0.9	0.194	0.554
unknown 7	3.08	-	(t)	-	-0.03	0.826	0.868
choline	3.19	56.69	(s)	3	0.22	0.026	0.306
[a]scyllo-inositol	3.35	76.4	(s)	3	0.05	0.726	0.868
taurine	3.42	38.07	(t)	4	0.28	0.081	0.488
acetoacetate	3.44	56.22	(s)	3	0.6	0.059	0.404
4-hydroxyphenylacetate (4-HPA)	3.44	46.34	(s)	4	0.6	0.059	0.404
glycine	3.56	44.18	(s)	3	-0.66	0.032	0.306
mannitol	3.86	65.94	(d)	4	0.07	0.74	0.868
mannitol	3.88	65.94	(d)	4	0.05	0.812	0.868
creatine	3.92	-	(s)	3	-0.12	0.644	0.859
[a]glycolate	3.94	64.32	(s)	3	-0.17	0.105	0.554
hippurate	3.96	46.46	(d)	4	-0.23	0.245	0.554
4-hydroxyhippuric acid	3.96	46.58	(d)	3	-0.23	0.245	0.554
tartrate	4.34	76.55	(s)	3	-0.15	0.507	0.737
unknown 8	6.07	-	(s)	-	-0.06	0.424	0.724
unknown 9	6.18	-	(s)	-	-0.27	0.404	0.724
fumarate	6.52	-	(s)	2	-0.16	0.256	0.554
4-hydroxyphenylacetate (4-HPA)	7.13	133.15	(d)	4	-0.14	0.85	0.868
hippurate	7.55	131.50	(t)	4	-0.37	0.275	0.554
hippurate	7.65	134.92	(m)	4	-0.35	0.276	0.554
[a]4-aminohippuric acid	7.67	133.02	(d)	3	0.09	0.472	0.724
indoxyl sulfate (IS)	7.70	127.07	(d)	3	-0.25	0.245	0.554
hippurate	7.83	129.85	(dd)	4	-0.33	0.335	0.644
hypoxanthine	8.18	148.27	(s)	3	-0.63	0.195	0.554
hypoxanthine	8.20	144.75	(s)	3	-0.1	0.849	0.868
formate	8.45	173.71	(s)	3	0.12	0.528	0.745
unknown 10	8.77	-	(d)	-	0.61	0.212	0.554
trigonelline	8.83	147.46	(t)	3	-0.14	0.748	0.868
trigonellinamide	8.89	-	(d)	2	-0.21	0.118	0.554
trigonellinamide	8.97	-	(d)	2	-0.2	0.15	0.554
trigonelline	9.11	148.50	(s)	3	-0.09	0.849	0.868
trigonellinamide	9.27	-	(s)	2	-0.23	0.165	0.554

unknown 11	9.36	-	(s)	-	-0.05	0.819	0.868

[a]Quantification may be unreliable because of spectral overlaps. Tentative assignment (Monteiro *et al* 2016) s=singlet, d=doublet, dd=doublet of doublet, m=multiplet. Fold change (FC) was calculated as the base 2 logarithm of the mean integral ratios between advanced and early RCC samples. Positive FC values indicate increased abundance in advanced RCC, while negative values indicate higher abundance in early RCC. *p*-values were calculated using the Student *T*-test, while *q*-values were computed by taking the FDR correction (Benjamini-Hochberg) after a Student *T*-test. Confidence score: (1) putatively characterized compound classes or annotated compounds, (2) matches from 1D NMR to literature and/or 1D BBiorefcode compound (AssureNMR) or other database libraries such as Biological Magnetic Resonance Bank (BMRB) and Human Metabolome Database (HMDB), (3) matched to Heteronuclear Single Quantum Coherence (HSQC), (4) matched to HSQC-TOCSY.

Table S2. RCC patient cohort characteristics for the 82 subjects used for tumor size predictions.

Characteristic	Number
**No of Urine Samples**	82
**Mean Age ± SD**	60.9 ± 13.1
**Mean BMI± SD**	29.3 ± 5.7
**Race**	
Caucasian	56
Black/African American	20
American-Indian/Alaskan- Native	1
Asian	1
Mixed	1
Unknown/Missing	3
**Smoker**	
Never	51
Former/Current	31
**Gender**	
Male	45
Female	36
Not Reported	1
**Histological Subtypes**	
Pure Clear Cell	57
Papillary	10
Clear Cell Papillary	6
Chromophobe	5
Unclassified	4
**Nuclear Grade**	
1	-
2	30
3	29
4	19
Unclassified	4
**RCC Stage**	
I	33
II	15
III	14
IV	8
Unclassified	12

Table S3. MS metabolomic features used in RCC stage stratification with p-values < 0.05 and > 1-fold change. Metabolite ID indicates name of metabolites or molecular weight underscore the retention time (MW_RT) of the metabolite.

ID	Metabolite ID	Formula	Mode	RT [min]	FC	T-test *p*-value
**50**	betaine	$C_5H_{11}NO_2$	positive	3.784	-1.04	0.02
**227**	O-desmethyltramadol	$C_{15}H_{23}NO_2$	positive	3.393	-4.94	0.08
**248**	195.11051_5.127		positive	5.127	2.31	< 0.001
**368**	oxybenzone	$C_{14}H_{12}O_3$	positive	1.483	-2.85	0.078
**628**	capuride	$C_9H_{18}N_2O_2$	positive	1.66	-1.74	0.039
**643**	386.07727_2.869	$C_{11}H_{24}N_4O_3P_2S_2$	positive	2.869	1.08	0.005
**776**	377.1422_1.733	$C_8H_{24}N_7O_8P$	positive	1.733	1.12	0.006
**782**	374.13924_1.753	$C_{17}H_{31}BrN_2S$	positive	1.753	1.14	0.004
**877**	411.18945_3.671		positive	3.671	-5.99	0.069
**919**	355.18568_2.993	$C_{13}H_{30}N_3O_6P$	positive	2.993	1.13	0.017
**963**	5-hydroxy-4-oxo-10-propyl-6,7,8,9-tetrahydro-4H-benzo[g]chromene-2-carboxylic acid	$C_{17}H_{18}O_5$	positive	3.023	-1.02	0.058
**1077**	(R)-1-aminopropan-2-ol	C_3H_9NO	positive	4.526	1.51	0.026
**1125**	75.03282_3.794		positive	3.794	-1.16	0.024
**1168**	N-acetylneuraminic acid	$C_{11}H_{19}NO_9$	positive	3.384	1.07	0.001
**1202**	447.21102_0.898	$C_{20}H_{33}NO_{10}$	positive	0.898	1.17	0.028
**1279**	N,N'-1,6-hexanediyldiacetamide	$C_{10}H_{20}N_2O_2$	positive	1.392	-1.91	0.02
**1372**	4-guanidinobutanoate	$C_5H_{11}N_3O_2$	positive	3.941	-1.12	0.004
**1536**	296.12631_1.171	$C_9H_{21}N_4O_5P$	positive	1.171	-1.81	0.015
**1542**	5-acetylamino-6-formylamino-3-methyluracil	$C_8H_{10}N_4O_4$	positive	1.749	1.36	0.004
**1543**	102.04408_1.751		positive	1.751	1.15	0.014
**1635**	73.06469_4.289		positive	4.289	1.1	0.004
**1673**	383.07001_0.804	$C_{15}H_{18}N_3O_5PS$	positive	0.804	1.15	0.017
**1689**	201.96195_4.715	$C_5H_5N_2OP_3$	positive	4.715	-1.36	0.042
**1718**	476.16636_1.696	$C_{18}H_{29}N_4O_9P$	positive	1.696	1.74	0.007
**1723**	202.09938_2.574	$C_6H_{14}N_6S$	positive	2.574	1.07	0.018
**1746**	394.13809_2.607	$C_{18}H_{22}N_2O_8$	positive	2.607	1.77	0.016
**1805**	341.20564_3.317	$C_9H_{28}N_9O_3P$	positive	3.317	5.02	0.003
**1904**	7-aminomethyl-7-carbaguanine	$C_7H_9N_5O$	positive	4.004	1.38	0.001
**1918**	476.16629_1.411	$C_{17}H_{33}O_{13}P$	positive	1.411	2.06	0.012
**1985**	265.16794_3.312		positive	3.312	-3.99	0.086
**2009**	chlortoluron	$C_{10}H_{13}ClN_2O$	positive	0.929	1.26	0.013
**2069**	471.21124_3.249	$C_{21}H_{34}N_3O_7P$	positive	3.249	1.14	0.009
**2085**	316.08436_1.77	$C_{11}H_{16}N_4O_5S$	positive	1.77	-1.07	0.049
**2113**	369.17887_0.903	$C_{12}H_{28}N_5O_6P$	positive	0.903	1.18	0.027
**2122**	Nalpha_Nalpha-dimethyl-L-histidine	$C_8H_{13}N_3O_2$	positive	1.209	1.12	0.001
**2176**	5-methyldeoxycytidine	$C_{10}H_{15}N_3O_4$	positive	0.761	1.14	0.013
**2178**	1-isothiocyanatobutane	C_5H_9NS	positive	1.268	-2.01	0.043
**2230**	77.03988_2.59		positive	2.59	1.09	0.016
**2242**	154.07975_2.587		positive	2.587	1.07	0.016
**2281**	214.16807_1.302		positive	1.302	-1.44	0.054
**2291**	2-amino-4-oxo-1,4,5,6,7,8-hexahydro-6-pteridinecarboxylic acid	$C_7H_9N_5O_3$	positive	1.387	1.01	0.013
**2313**	331.08835_3.342	$C_{10}H_{24}NO_5P_3$	positive	3.342	1.04	0.002
**2317**	diethyl2-methyl-3-oxosuccinate	$C_9H_{14}O_5$	positive	0.892	1.51	0.019
**2329**	gabapentin	$C_9H_{17}NO_2$	positive	1.078	-1.48	0.05
**2339**	220.0483_2.073	$C_4H_9N_6O_3P$	positive	2.073	-1.21	0.071
**2377**	576.20709_1.722	$C_{25}H_{36}O_{15}$	positive	1.722	-1.07	0.049
**2381**	coumarin	$C_9H_6O_2$	positive	1.184	-2.21	0.061
**2417**	529.97494_4.637	$C_{11}H_{21}N_2O_{12}P_3S_2$	positive	4.637	-1.17	0.053

**2440**	354.19077_3.015	$C_{10} H_{27} N_8 O_4 P$	positive	3.015	1.13	0.011
**2465**	3-hydroxyanthranilic acid	$C_7 H_7 N O_3$	positive	0.893	1.41	0.005
**2532**	249.17283_1.127	$C_9 H_{24} N_5 O P$	positive	1.127	-5.18	0.081
**2553**	304.19036_3.455	$C_{14} H_{29} N_2 O_3 P$	positive	3.455	-1.14	0.064
**2558**	236.05433_3.255	$C_9 H_{18} O P_2 S$	positive	3.255	1.12	0.006
**2583**	278.11567_1.159	$C_{10} H_{15} N_8 P$	positive	1.159	-2.06	0.013
**2601**	119.0499_2.658	$C_4 H_{10} N O P$	positive	2.658	1.05	0.028
**2618**	cyclamic acid	$C_6 H_{13} N O_3 S$	positive	4.026	1.43	0.006
**2663**	229.03739_2.667	$C_4 H_{13} N_3 O_4 P_2$	positive	2.667	1.49	0.013
**2738**	322.14655_2.209	$C_{13} H_{27} N_2 O_3 P S$	positive	2.209	-1.11	0.072
**2817**	259.12077_0.59	$C_9 H_{18} N_5$ O2 P	positive	0.59	1.77	0.022
**2877**	476.16623_1.613	$C_{23} H_{25} N_8 P S$	positive	1.613	1.52	0.012
**2905**	122.05326_2.579		positive	2.579	1.24	0.006
**2932**	122.05327_2.649		positive	2.649	1.24	0.009
**2934**	118.05734_3.808		positive	3.808	-1.3	0.001
**3001**	494.21598_0.893	$C_{17} H_{40} N_2 O_{10} P_2$	positive	0.893	1.39	0.018
**3093**	239.05433_3.208	$C_9 H_9 N_3 O_5$	positive	3.208	1.09	0.005
**3109**	clavulanic acid	$C_8 H_9 N O_5$	positive	1.103	1.48	0.004
**3149**	561.17146_1.483	$C_{18} H_{36} N_5 O_9 P S_2$	positive	1.483	-1.78	0.049
**3163**	278.1117_3.53	$C_5 H_{15} N_{10} O_2 P$	positive	3.53	1.53	< 0.001
**3191**	593.87057_4.791	$C_6 H_6 Cl_2 N_8 O_{17} P_2$	positive	4.791	-1.96	0.07
**3193**	845.49958_0.797	$C_{46} H_{78} N_3 O_3 P_3 S$	positive	0.797	1.75	0.017
**3257**	258.06759_3.675		positive	3.675	-1.94	0.029
**3262**	577.89669_4.751	$C_{10} H_{14} Cl N_2 O_{16} P_3 S$	positive	4.751	-1.88	0.014
**3297**	471.93285_4.706	$C_{11} H_6 N_8 O_6 P_2 S_2$	positive	4.706	-1.28	0.056
**3306**	459.14964_2.694	$C_{12} H_{26} N_7 O_{10} P$	positive	2.694	1.3	0.011
**3370**	553.93601_4.703	$C_8 H_{13} N_8 O_{11} P_3 S_2$	positive	4.703	-1.23	0.062
**3405**	259.09186_1.132	$C_8 H_{13} N_5 O_5$	positive	1.132	-1.29	0.016
**3498**	282.06749_0.762	$C_{12} H_{16} N_2 O_2 P_2$	positive	0.762	-1.26	0.07
**3574**	279.06783_0.721	$C_6 H_{14} N_7 O_2 P S$	positive	0.721	-1.34	0.048
**3597**	254.12687_3.235		positive	3.235	1.61	0.014
**3602**	4-ethylguaiacol	$C_9 H_{12} O_2$	positive	0.886	1.01	0.022
**3719**	471.17321_2.498	$C_{18} H_{31} N_7 O_2 P_2 S$	positive	2.498	1.04	0.022
**3746**	momilactoneA	$C_{20} H_{26} O_3$	positive	0.915	1.13	0.01
**3766**	apo-[3-methylcrotonoyl-CoA:carbon-dioxide ligase (ADP-forming)]	$C_7 H_{15} N_3 O_2$	positive	3.633	1.04	0.001
**3857**	379.13545_1.855	$C_{11} H_{22} N_7 O_6 P$	positive	1.855	1.07	0.014
**3934**	8-azabicyclo[3.2.1]octan-3-ol	$C_7 H_{13} N O$	positive	4.194	1.45	0.023
**3943**	2-amino-6-[(2,5-dihydroxy-2-oxido-1,3,2-dioxaphos-phinan-4-yl)(hydroxy)methyl]-4a,5,8,8a-tetrahydro-4(3H)-pteridinone	$C_{10} H_{16} N_5 O_7 P$	positive	0.91	1.57	0.008
**4050**	487.907_4.72	$C_4 H_{11} N_8 O_8 P_3 S_3$	positive	4.72	-1.18	0.061
**4090**	301.0258_1.138	$C_5 H_{14} N_5 O_4 P_3$	positive	1.138	-1.8	0.035
**4097**	230.16336_3.107		positive	3.107	1.55	0.018
**4116**	118.05095_3.799		positive	3.799	-1.24	0.001
**4120**	376.18896_1.041	$C_{14} H_{30} N_6 O_2 P_2$	positive	1.041	-1.19	0.076
**4127**	Dyphylline	$C_{10} H_{14} N_4 O_4$	positive	3.812	-1.49	0.064
**4145**	N_N-dihydroxy-L-tyrosine	$C_9 H_{11} N O_5$	positive	3.529	-2.45	0.007
**4259**	791.95788_4.622	$C_{17} H_{27} N_6 O_{16} P_3 S_4$	positive	4.622	-1.11	0.01
**4287**	874.35494_3.594	$C_{35} H_{54} N_8 O_{18}$	positive	3.594	1.14	0.02
**4291**	93.0254_4.027		positive	4.027	1.29	0.006
**4391**	139.03566_3.806	$C H N_9$	positive	3.806	-1.49	0.002
**4393**	294.11955_3.243	$C_8 H_{19} N_6 O_4 P$	positive	3.243	1.53	0.012
**4460**	453.11034_3.314	$C_{13} H_{25} N_7 O_5 P_2 S$	positive	3.314	-2.92	0.018
**4467**	Glycine	$C_2 H_5 N O_2$	positive	3.787	-1.02	0.041

**4569**	182.08223_4.719	$C_5 H_{15} N_2 O_3 P$	positive	4.719	-1.13	0.06
**4702**	4-[(2-cyclohex-1-enylethyl)amino]-4-oxobut-2-enoic acid	$C_{12} H_{17} N O_3$	negative	2.567	-1.02	0.062
**4836**	94.05175_4.022		negative	4.022	-1.07	0.069
**4902**	394.09177_0.894	$C_{10} H_{19} N_8 O_5 P S$	negative	0.894	-1.48	0.068
**4938**	271.05123_0.934	$C_6 H_{10} N_9 P S$	negative	0.934	1.36	0.005
**4947**	2-hydroxymethylserine	$C_4 H_9 N O_4$	negative	2.368	-1.22	0.02
**4948**	230.08767_0.692	$C_5 H_{10} N_8 O_3$	negative	0.692	1.17	0.014
**4992**	1058.43938_3.7	$C_{47} H_{68} N_{10} O_{14} P_2$	negative	3.7	1.61	0.01
**5045**	219.01948_3.496	$C_7 H_9 N O_5 S$	negative	3.496	1.03	0.002
**5065**	427.01281_0.855	$C_{11} H_{15} Cl N_5 O_7 P S$	negative	0.855	1.58	< 0.001
**5087**	215.03023_3.824	$C_3 H_{10} N_3 O_6 P$	negative	3.824	-1.78	0.028
**5127**	128.04606_0.939		negative	0.939	1.32	0.008
**5192**	272.13713_3.256	$C_{12} H_{20} N_2 O_5$	negative	3.256	1.36	0.007
**5206**	874.35426_3.608	$C_{36} H_{65} N_2 O_{16} P_3$	negative	3.608	1.15	0.022
**5226**	175.02321_0.931	$C_4 H_5 N_3 O_5$	negative	0.931	1.17	0.023
**5249**	347.03427_3.525	$C_{10} H_{13} N_5 O_5 S_2$	negative	3.525	1.51	0.002
**5255**	241.04429_3.202	$C_7 H_7 N_5 O_5$	negative	3.202	-1.9	0.005
**5379**	283.04235_0.863	$C_{12} H_{14} N O_3 P S$	negative	0.863	1.15	0.001
**5406**	358.07601_3.191	$C_{12} H_{24} O_6 P_2 S$	negative	3.191	1.43	0.026
**5408**	1-(beta-D-ribofuranosyl)-1,2-dihydropyrimidine	$C_9 H_{14} N_2 O_4$	negative	3.076	1.17	< 0.001
**5409**	585.08288_0.867	$C_{17} H_{34} Cl_2 N_5 O_5 P S_3$	negative	0.867	1.28	0.003
**5417**	168.00246_3.362	$C_4 H_{11} O P_3$	negative	3.362	-1.11	0.047
**5420**	206.06051_3.38	$C_4 H_{12} N_6 P_2$	negative	3.38	1.73	0.003
**5437**	124.01817_0.764		negative	0.764	2.18	< 0.001
**5448**	407.07324_3.497	$C_{11} H_{21} N O_{13} S$	negative	3.497	1.09	0.001
**5481**	91.99188_3.515		negative	3.515	1.34	0.002
**5482**	589.07516_0.858	$C_{23} H_{29} N O_9 P_2 S_2$	negative	0.858	1.7	0.001
**5485**	318.12476_3.497	$C_{13} H_{22} N_2 O_5 S$	negative	3.497	-1.3	0.047
**5511**	251.06244_3.507	$C_6 H_{16} N_5 P_3$	negative	3.507	1.33	0.005
**5518**	(1R_2S)-1-hydroxypropane-1_2_3-tricarboxylate	$C_6 H_8 O_7$	negative	3.819	-2.08	0.034
**5546**	430.06125_3.828		negative	3.828	-2.13	0.033
**5580**	333.9991_3.386	$C_8 H_{12} N_6 O P_2 S_2$	negative	3.386	-2.83	0.067
**5626**	91.99188_3.433		negative	3.433	1.13	0.009
**5636**	382.07789_0.946	$C_6 H_{20} N_6 O_9 P_2$	negative	0.946	-1.66	0.045
**5666**	294.96983_0.848	$C_7 H_{12} N_3 P_3 S_2$	negative	0.848	-3.21	0.072
**5680**	361.08868_3.981	$C_9 H_{20} N_3 O_{10} P$	negative	3.981	1.06	0.012
**5713**	306.10614_1.236	$C_{11} H_{18} N_2 O_8$	negative	1.236	1.02	0.016
**5729**	528.03813_3.824	$C_6 H_{21} N_6 O_{18} P S$	negative	3.824	-1.7	0.017
**5737**	gly-Lys	$C_8 H_{17} N_3 O_3$	negative	3.995	1.14	0.001
**5785**	472.19426_3.42	$C_{15} H_{34} N_6 O_7 P_2$	negative	3.42	1.61	0.013
**5813**	193.02945_0.927	$C_9 H_8 N O_2 P$	negative	0.927	1.17	0.008
**5825**	313.00756_3.83	$C_4 H_9 N_7 O_6 P_2$	negative	3.83	-1.49	0.019
**5871**	508.96107_3.835	$C_6 H_{10} N_9 O_{13} P_3$	negative	3.835	-1.28	0.027
**5876**	183.03078_0.642	$C_6 H_6 Cl N_5$	negative	0.642	1.24	0.004
**5898**	404.15254_4.243	$C_{13} H_{27} N_8 O P_3$	negative	4.243	1.52	0.005
**5912**	243.11029_3.464	$C_{11} H_{17} N O_5$	negative	3.464	1.21	0.008
**5985**	succinic anhydride	$C_4 H_4 O_3$	negative	0.944	1.53	0.006
**6069**	138.06677_2.05		negative	2.05	-1.24	0.019
**6089**	tribenuron-methyl [ANSI]	$C_{15} H_{17} N_5 O_6 S$	negative	0.862	-1.83	0.063
**6124**	114.03038_0.931		negative	0.931	1.07	0.019
**6256**	metsulfuron-methyl	$C_{14} H_{15} N_5 O_6 S$	negative	0.889	-1.77	0.034
**6337**	369.05388_0.862	$C_{10} H_{12} N_9 O_3 P S$	negative	0.862	1.34	< 0.001
**6351**	190.04676_0.934		negative	0.934	1.12	0.02
**6396**	367.05878_0.884	$C_{11} H_{18} N_3 O_7 P S$	negative	0.884	1.3	< 0.001

**6428**	75.03065_3.812		negative	3.812	-1.2	0.011
**6458**	130.02558_0.93		negative	0.93	1.06	0.028
**6466**	ecgoninemethylester	$C_{10} H_{17} N O_3$	negative	2.657	-1.06	0.077
**6683**	203.04083_3.025	$C_{11} H_9 N O S$	negative	3.025	-1.1	0.027
**6687**	370.05739_0.866	$C_6 H_{14} N_{10} O_5 S_2$	negative	0.866	1.33	< 0.001
**6694**	410.98437_3.827	$C_2 H_5 N_9 O_{16}$	negative	3.827	-1.3	0.019
**6719**	329.07459_2.713	$C_{10} H_{17} N_7 P_2 S$	negative	2.713	1.44	0.025
**6731**	370.0522_0.859		negative	0.859	1.1	0.004
**6738**	118.0253_0.926		negative	0.926	1.17	0.007
**6787**	132.04102_0.939		negative	0.939	1.07	0.01
**6835**	249.06673_3.522	$C_9 H_{15} N O_5 S$	negative	3.522	1.08	0.004
**6912**	117.05251_3.817		negative	3.817	-1.63	< 0.001
**6933**	194.03025_3.794	$C_4 H_2 N_8 O_2$	negative	3.794	-1.17	0.038
**6952**	411.18928_3.694	$C_{15} H_{26} N_9 O_3 P$	negative	3.694	-5.43	0.073
**6980**	412.10235_0.884	$C_{10} H_{21} N_8 O_6 P S$	negative	0.884	-1.54	0.057
**6994**	455.21558_3.668	$C_{22} H_{33} N O_9$	negative	3.668	-7.01	0.073
**7001**	371.04981_0.869	$C_{11} H_{15} N_7 O_2 P_2 S$	negative	0.869	1.35	< 0.001

Table S4. Machine learning prediction results for individual metabolites in the RCC staging metabolomic panel. Results are presented as mean +/- standard deviation of a 10-fold cross validation classification.

Metabolite Identity or ID	AUC	Accuracy	Sensitivity	Specificity
**4-guanidinobutanoic acid**	0.73 +/- 0.2	0.66 +/- 0.13	0.5 +/- 0.34	0.78 +/- 0.23
**7-aminomethyl-7-carbaguanine**	0.73 +/- 0.19	0.69 +/- 0.17	0.5 +/- 0.31	0.8 +/- 0.19
**N,N- dimethyl- histidine**	0.79 +/- 0.2	0.69 +/- 0.12	0.6 +/- 0.25	0.76 +/- 0.27
**diethyl-2-methyl-3-oxosuccinate**	0.59 +/- 0.24	0.54 +/- 0.19	0.27 +/- 0.25	0.72 +/- 0.24
**3-hydroxyanthranilic acid**	0.52 +/- 0.18	0.6 +/- 0.11	0.37 +/- 0.18	0.76 +/- 0.16
**cytosine dimer**	0.66 +/- 0.24	0.63 +/- 0.15	0.43 +/- 0.33	0.76 +/- 0.18
**3163**	0.72 +/- 0.19	0.74 +/- 0.11	0.53 +/- 0.27	0.88 +/- 0.16
**apo-[3-methylcrotonoyl-CoA:carbon-dioxide ligase (ADP-forming)]**	0.74 +/- 0.21	0.73 +/- 0.16	0.48 +/- 0.37	0.9 +/- 0.12
**4116**	0.76 +/- 0.16	0.64 +/- 0.17	0.52 +/- 0.16	0.73 +/- 0.26
**5045**	0.79 +/- 0.12	0.73 +/- 0.1	0.67 +/- 0.26	0.78 +/- 0.16
**dihydrouridine**	0.5 +/- 0.11	0.44 +/- 0.16	0.25 +/- 0.23	0.58 +/- 0.2
**5420**	0.72 +/- 0.19	0.67 +/- 0.09	0.45 +/- 0.26	0.82 +/- 0.16
**5437**	0.61 +/- 0.21	0.63 +/- 0.13	0.35 +/- 0.26	0.83 +/- 0.16
**5713**	0.63 +/- 0.22	0.6 +/- 0.2	0.37 +/- 0.35	0.76 +/- 0.18
**lys-gly/ gly-lys**	0.83 +/- 0.16	0.67 +/- 0.16	0.53 +/- 0.27	0.78 +/- 0.2
**succinic anhydride**	0.68 +/- 0.19	0.69 +/- 0.15	0.48 +/- 0.26	0.82 +/- 0.16
**hydroxyprolyl-asparagine**	0.61 +/- 0.23	0.57 +/- 0.11	0.27 +/- 0.2	0.78 +/- 0.13
**6687**	0.63 +/- 0.27	0.7 +/- 0.13	0.47 +/- 0.22	0.86 +/- 0.16
**6694**	0.57 +/- 0.19	0.57 +/- 0.2	0.47 +/- 0.31	0.66 +/- 0.2
**citrate**	0.65 +/- 0.18	0.56 +/- 0.13	0.35 +/- 0.22	0.7 +/- 0.22
**glycine**	0.72 +/- 0.2	0.69 +/- 0.17	0.57 +/- 0.3	0.78 +/- 0.24
**choline**	0.49 +/- 0.12	0.57 +/- 0.11	0.3 +/- 0.28	0.75 +/- 0.22
**acetone**	0.61 +/- 0.18	0.67 +/- 0.13	0.48 +/- 0.26	0.8 +/- 0.15
**pyruvate**	0.53 +/- 0.2	0.57 +/- 0.17	0.35 +/- 0.22	0.72 +/- 0.24

Table S5. Demographics/clinical information for healthy controls cohort.

Characteristic	Number
**No of Urine Samples**	174
**Mean Age ± SD**	54.4 ± 10.32
**Mean BMI± SD**	27.3 ± 4.52
**Race**	
Caucasian	162(93.1%)
Black/African American	5(2.9%)
Others	7(4.0%)
**Smoker**	
Never	131(75.3%)
Former/Current	43(24.7%)
**Gender**	
Male	145(83.3%)
Female	29(16.6%)

Table S6. RCC staging markers that are statistically significant (Welch's *T*-test $p < 0.05$) when RCC (n=82) was compared with the healthy controls (n=176). Fold change (FC) was calculated as the base 2 logarithm of the average intensity ratios between RCC and controls samples. Positive FC values indicate increased abundance in RCC, while negative values indicate higher abundance in control samples.

Metabolite or ID	FC	*p*-values	*q*-values	Increased in RCC (*vs.* Controls)	Increase in Advanced RCC (*vs.* Early RCC)
**6687**	-1.86	2.39E-14	5.73E-13	No	Yes
**lys-gly/ gly-lys**	1.26	1.15E-10	1.37E-09	Yes	Yes
**7-aminomethyl-7-carbaguanine**	1.32	5.17E-08	4.14E-07	Yes	Yes
**hydroxyprolyl-asparagine/ asparaginylhydroxyproline**	0.91	7.85E-07	4.71E-06	Yes	Yes
**glycine**	0.57	1.63E-06	7.80E-06	Yes	No
**pyruvate**	0.22	6.36E-06	2.54E-05	Yes	Yes
**acetone**	0.38	7.68E-06	2.63E-05	Yes	Yes
**5420**	1.10	3.17E-04	9.50E-04	Yes	Yes
**citrate**	0.28	1.56E-03	4.16E-03	Yes	No
**apo-[3-methylcrotonoyl-CoA:carbon-dioxide ligase (ADP-forming)]**	0.46	2.09E-03	5.01E-03	Yes	Yes
**6694**	-1.25	2.60E-03	5.66E-03	No	No
**dihydrouridine**	0.50	1.08E-02	2.15E-02	Yes	Yes
**3163**	0.42	3.65E-02	6.74E-02	Yes	Yes

Table S7. Metabolomic features identified as statistically significant (Welch's T-test, BH $q < 0.05$; Log2 FC > 2) when healthy controls (n=176) were compared with early stage RCC (n=41). Fold change (FC) was calculated as the base 2 logarithm of the average intensity ratios between early RCC and controls samples. Positive FC values indicate increased abundance in early RCC, while negative values indicate higher abundance in control samples.

Metabolite ID/Tentative ID	Molecular Weight	Mode	RT [min]	Formula	FC	T-test *p*-value	FDR *p*-value
**37**	235.176	positive	0.893	-	7.68	0.012	0.04539
**52**	264.19665	positive	3.806	$C_{12} H_{29} N_2 O_2 P$	10.98	0.002	0.013476
**114**	528.39173	positive	3.84	$C_{32} H_{52} N_2 O_4$	10.52	0.005	0.023978
**272**	90.0469	positive	1.003	$C_7 H_6$	5.09	0.00006	0.000696
**292/2-(3-Phenylpropyl)pyridine**	197.12052	positive	1	$C_{14} H_{15} N$	6.41	0.00006	0.00072
**442**	514.00051	positive	4.64	$C_{10} H_{12} N_8 O_{13} P_2$	2.07	0.0062	0.028968
**698/ Dibenzylamine**	90.04688	positive	0.931	$C_{14} H_{15} N$	5.48	0.00026	0.002287
**703/ Dibenzylamine**	197.12036	positive	0.932	$C_{14} H_{15} N$	6.85	0.00026	0.002277
**743**	477.14347	positive	2.506	$C_{17} H_{28} N_5 O_7 P S$	2.58	0.01	0.044628
**901**	250.16823	positive	3.145	NaN	9.38	0.0004	0.003413
**1002**	283.96514	positive	4.677	$C_5 H_3 N_8 O P_3$	3.17	0.0048	0.023696
**1003**	283.9651	positive	4.731	$C_5 H_3 N_8 O P_3$	3.16	0.0050	0.024606
**1191**	374.15553	positive	2.404	$C_{14} H_{33} O_5 P_3$	4.06	0.0042	0.021302
**1205**	166.06638	positive	4.75	-	6.60	0.000005	0.000081
**1308**	90.04757	positive	1.003	-	2.94	0.000013	0.000207
**1563**	517.10583	positive	3.009	$C_{19} H_{26} Cl N_5 O_6 P_2$	6.44	0.003407	0.018114
**1662**	370.1313	positive	2.502	$C_{13} H_{27} N_2 O_6 P S$	2.12	0.010333	0.043946
**1689**	201.96195	positive	4.715	$C_5 H_5 N_2 O P_3$	2.23	0.000946	0.006493
**1814**	211.01244	positive	0.861	$C_9 H_9 N O S_2$	8.13	0.000008	0.000135
**1840**	241.14653	positive	0.845	NaN	4.75	0.000011	0.00018
**1899**	325.07842	positive	1.26	$C_{11} H_{21} Cl N_3 O_2 P S$	3.61	0.002377	0.013644
**2175**	503.3166	positive	0.655	$C_{29} H_{46} N O_4 P$	6.32	0.006032	0.028618
**2307**	486.29021	positive	0.646	$C_{29} H_{43} O_4 P$	6.21	0.005942	0.028286
**2417**	529.97494	positive	4.637	$C_{11} H_{21} N_2 O_{12} P_3 S_2$	2.28	0.000615	0.004658
**2448**	529.97474	positive	4.712	$C_9 H_{16} N_4 O_{16} P_2 S$	3.17	0.001211	0.007978
**2523**	307.92638	positive	4.679	$C_5 H_3 N_4 O_6 P_3$	3.26	0.012166	0.049958
**2540**	463.94578	positive	4.732	$C_7 H_9 N_6 O_{12} P S_2$	3.09	0.000836	0.005911
**2887**	519.10301	positive	3.011	$C_{23} H_{21} N O_{13}$	4.37	0.003868	0.020051

**2924**	545.94887	positive	4.727	$C_7 H_{17} N_8 O_9 P_3 S_3$	3.04	0.000872	0.006093
**3025**	611.978	positive	4.7	$C_{19} H_{15} N_6 O_{10} P_3 S$	2.43	0.000516	0.004046
**3168**	234.10682	positive	0.904	NaN	3.48	0.011811	0.048892
**3246**	327.07559	positive	1.266	$C_9 H_{19} N_3 O_6 P_2$	4.28	0.00288	0.01571
**3262**	577.89669	positive	4.751	$C_{10} H_{14} Cl N_2 O_{16} P_3 S$	2.50	0.000698	0.005171
**3297**	471.93285	positive	4.706	$C_{11} H_6 N_8 O_6 P_2 S_2$	2.70	0.000604	0.004604
**3370**	553.93601	positive	4.703	$C_8 H_{13} N_8 O_{11} P_3$ S2	2.64	0.000658	0.004921
**3564**	414.20445	positive	0.848	$C_{18} H_{31} N_4 O_5 P$	3.47	0.000003	0.000063
**3620**	693.9807	positive	4.72	$C_{16} H_{12} N_{10} O_{18} P_2$	3.55	0.006049	0.02864
**3643**	413.20506	positive	0.858	$C_{15} H_{38} N_5 P_3 S$	2.69	0.000033	0.000447
**3761**	635.93901	positive	4.722	$C_{11} H_{27} O_{16} P_3 S_4$	3.914426	0.009177	0.039912
**3976**	569.91008	positive	4.73	$C_9 H_{10} Cl N_6 O_{15} P_3$	3.25	0.001535	0.009635
**3998**	168.06169	positive	4.722	$C_2 H_4 N_{10}$	3.07	0.000019	0.000274
**4015**	421.87763	positive	4.748	$C_4 H_6 O_{17} S_3$	2.59	0.000464	0.003682
**4050**	487.907	positive	4.72	$C_4 H_{11} N_8 O_8 P_3 S_3$	2.37	0.000565	0.004354
**4145/ N_N-Dihydroxy-L-tyrosine**	213.06359	positive	3.529	$C_9 H_{11} N O_5$	2.14	0.0029	0.015768
**4283**	799.94579	positive	4.687	$C_{19} H_{19} N_{10} O_{14} P_3 S_3$	2.02	0.000382	0.003137
**4626/2-Naphthalenesulfonic acid**	208.01858	negative	0.66	$C_{10} H_8 O_3 S$	3.26	0.000058	0.000672
**5010**	673.97808	negative	4.637	$C_{12} H_{21} N_8 O_{15} P_3 S_2$	2.02	0.000008	0.000134
**5078**	357.99984	negative	3.606	$C_7 H_{13} N_4 O_7 P_3$	2.71	0.000068	0.000756
**5285**	650.01659	negative	4.644	$C_{18} H_{20} N_8 O_{11} P_2 S_2$	2.60	0.000024	0.000332
**5310**	394.28488	negative	0.87	$C_{21} H_{43} Cl O_4$	4.18	0.0005	0.003941
**5936**	372.17823	negative	1.327	$C_{18} H_{28} O_8$	6.98	0.002581	0.014546
**6456**	370.16253	negative	1.413	$C_{19} H_{22} N_4 O_4$	2.65	0.010058	0.043058
**6578**	166.98523	negative	0.832	$C_6 H N O_5$	3.37	0.000011	0.000177
**6740**	356.00096	negative	3.607	$C_7 H_{14} Br N_6 O_4 P$	2.74	0.000104	0.001065
**7057**	648.43521	negative	3.788	$C_{32} H_{56} N_8 O_6$	5.21	0.007612	0.034278

Table S8. Comparison of RCC staging markers in early staged RCC (*n*=41) *vs.* healthy controls (*n* = 176). Fold change (FC) was calculated as the base 2 logarithm of the average intensity ratios between early-staged RCC and controls samples. Positive FC values indicate increased abundance in early-staged RCC, while negative values indicate higher abundance in control samples. P-values are computed using Welch's *T*-test, and *q*-values represent Benjamini-Hochberg correction. The first 13 metabolomic features has *p* values less than 0.05.

Metabolite/ID	FC	*p*-value	*q*-value	Increase in Early RCC (*vs.* Controls)	Increase in Advanced RCC (*vs.* Early RCC)
**6687**	-2.53	3.78E-26	1.36E-23	No	Yes
**5437**	-1.67	1.04E-07	2.96E-06	No	Yes
**citrate**	0.39	1.33E-03	8.62E-03	Yes	No
**6694**	-1.02	1.69E-03	1.04E-02	No	No
**choline**	-0.21	2.67E-03	1.48E-02	No	Yes
**glycine**	0.82	2.73E-03	1.51E-02	Yes	No
**3-hydroxyanthranilic acid**	-0.87	3.88E-03	2.01E-02	No	Yes
**5045**	-1.05	6.27E-03	2.94E-02	No	Yes
**cytosine dimer**	-0.41	6.48E-03	3.02E-02	No	Yes
**lys-gly/ gly-lys**	0.73	1.11E-02	4.64E-02	Yes	Yes
**7-aminomethyl-7-carbaguanine**	0.69	1.67E-02	6.42E-02	Yes	Yes
**5713**	-0.49	3.06E-02	1.03E-01	No	Yes
**hydroxyprolyl-asparagine/ asparaginylhydroxyproline**	0.5	3.16E-02	1.05E-01	Yes	Yes
**pyruvate**	0.09	6.50E-02	1.75E-01	Yes	Yes
**4-guanidinobutanoic acid**	0.49	8.05E-02	2.03E-01	Yes	No
**diethyl-2-methyl-3-oxosuccinate**	-0.82	1.09E-01	2.50E-01	No	Yes
**succinic anhydride**	-0.5	1.10E-01	2.51E-01	No	Yes
**acetone**	0.16	1.75E-01	3.39E-01	Yes	Yes
**3163**	-0.36	2.22E-01	3.94E-01	No	Yes
**N,N- dimethyl- histidine**	-0.24	3.18E-01	4.87E-01	No	Yes
**dihydrouridine**	0.22	3.62E-01	5.19E-01	Yes	Yes
**5420**	0.22	6.04E-01	7.31E-01	Yes	Yes
**4116**	-0.09	6.43E-01	7.60E-01	No	No
**apo-[3-methylcrotonoyl-CoA:carbon-dioxide ligase (ADP-forming)]**	0.01	9.48E-01	9.67E-01	Yes	Yes

Table S9. Metabolomic features identified as statistically significant (Welch's T-test, BH *q* < 0.05; Log2 FC > 2) when healthy controls (*n*=176) were compared with advanced stage RCC (*n*=29). Fold change (FC) was calculated as the base 2 logarithm of the average intensity ratios between advanced stage RCC and controls samples. Positive FC values indicate increased abundance in advanced RCC, while negative values indicate higher abundance in control samples.

Metabolite ID/Tentative ID	Molecular Weight	Mode	RT [min]	Formula	FC	T-test p-value	FDR p-value
**37**	235.176	positive	0.893	-	7.99	0.004928	0.026213
**52**	264.19665	positive	3.806	$C_{12}H_{29}N_2O_2P$	11.53	0.003551	0.019884
**114**	528.39173	positive	3.84	$C_{32}H_{52}N_2O_4$	11.25	0.003694	0.020526
**128**	225.10011	positive	2.99	-	2.31	0.004682	0.02515
**132**	121.0634	positive	2.975	-	2.20	0.004664	0.025075
**199**	256.14218	positive	2.952	-	2.01	0.010085	0.044754
**901**	250.16823	positive	3.145	-	8.68	0.010218	0.045224
**919**	355.18568	positive	2.993	$C_{13}H_{30}N_3O_6P$	2.51	0.001831	0.011842
**995**	85.08981	positive	0.882	-	3.73	0.005829	0.029834
**1112**	235.17125	positive	0.896	-	6.65	0.005951	0.030392
**1205**	166.06638	positive	4.75	-	6.99	0.002139	0.013432
**1216**	369.20141	positive	2.947	$C_{14}H_{32}N_3O_6P$	2.16	0.008023	0.037788
**1724**	341.20527	positive	3.36	$C_9H_{28}N_9O_3P$	6.37	0.01077	0.047065
**1814**	211.01244	positive	0.861	$C_9H_9NOS_2$	8.68	0.000771	0.005944
**1840**	241.14653	positive	0.845	NaN	5.64	0.001435	0.009828
**1899**	325.07842	positive	1.26	$C_{11}H_{21}ClN_3O_2PS$	3.55	0.003455	0.019517
**1904/7-aminomethyl-7-carbaguanine**	179.08065	positive	4.004	$C_7H_9N_5O$	2.07	0.00095	0.007013
**1938**	385.23167	positive	3.407	$C_{16}H_{35}NO_9$	6.17	0.006926	0.034012
**2282**	383.21641	positive	3.251	$C_{17}H_{29}N_5O_5$	4.87	0.005686	0.029332
**2292**	250.16803	positive	0.915	NaN	3.44	0.009524	0.042715
**2328**	266.12425	positive	3.014	$C_9H_{14}N_8O_2$	2.34	0.000504	0.00418
**2365**	390.18696	positive	3.407	$C_{12}H_{31}N_4O_8P$	5.51	0.008905	0.040498
**2440**	354.19077	positive	3.015	$C_{10}H_{27}N_8O_4P$	2.09	0.002099	0.013227
**2557**	427.24242	positive	3.304	$C_{19}H_{33}N_5O_6$	3.80	0.003473	0.019571
**2618/ Cyclamic acid**	179.06157	positive	4.026	$C_6H_{13}NO_3S$	2.01	0.00451	0.024367
**2632**	429.25808	positive	3.445	$C_{13}H_{36}N_9O_5P$	5.91	0.008617	0.039622
**2715**	465.1362	positive	1.885	$C_{17}H_{23}N_9O_3S_2$	2.05	0.000456	0.003838
**2779**	388.17127	positive	3.251	$C_{15}H_{35}O_5P_3$	5.36	0.006094	0.030904
**2893**	86.09313	positive	0.896	-	3.78	0.004878	0.026028
**3168**	234.10682	positive	0.904	-	3.87	0.005346	0.027963

**3246**	327.07559	positive	1.266	$C_9 H_{19} N_3 O_6 P_2$	4.07	0.004079	0.022385
**3292**	471.2686	positive	3.351	$C_{24} H_{43} N O_4 P_2$	5.57	0.003264	0.018655
**3447**	432.19778	positive	3.307	$C_{15} H_{32} N_2 O_{12}$	4.63	0.002619	0.015727
**3556**	589.2953	positive	3.224	$C_{23} H_{48} N_3 O_{12} P$	5.09	0.010424	0.045747
**3564**	414.20445	positive	0.848	$C_{18} H_{31} N_4 O_5 P$	2.93	0.000734	0.005731
**3597**	254.12687	positive	3.235	-	2.58	0.007032	0.034432
**3643**	413.20506	positive	0.858	$C_{15} H_{38} N_5 P_3 S$	2.16	0.00232	0.014288
**4064**	302.19543	positive	3.018	$C_{13} H_{26} N_4 O_4$	2.51	0.00129	0.008959
**4134**	547.2848	positive	3.337	$C_{21} H_{46} N_3 O_{11} P$	5.65	0.005499	0.028546
**4445**	552.24012	positive	3.34	$C_{17} H_{40} N_6 O_{12} S$	4.82	0.004168	0.022765
**4626/2-Naphthalenesulfonic acid**	208.01858	negative	0.66	$C_{10} H_8 O_3 S$	4.10	0.009225	0.041559
**4992**	1058.43938	negative	3.7	$C_{47} H_{68} N_{10} O_{14} P_2$	2.98	0.002867	0.016886
**5078**	357.99984	negative	3.606	$C_7 H_{13} N_4 O_7 P_3$	3.15	0.000384	0.003369
**5285**	650.01659	negative	4.644	$C_{18} H_{20} N_8 O_{11} P_2 S_2$	2.08	0.001454	0.009935
**5310**	394.28488	negative	0.87	$C_{21} H_{43} Cl O_4$	3.35	0.002964	0.017222
**5642/1-(4-Amino-4-carboxybutanoyl)-2-piperidinecarboxylic acid**	258.1213	negative	3.297	$C_{11} H_{18} N_2 O_5$	2.17	0.008633	0.039667
**5912**	243.11029	negative	3.464	$C_{11} H_{17} N O_5$	2.16	0.00175	0.011461
**5936**	372.17823	negative	1.327	$C_{18} H_{28} O_8$	7.25	0.010831	0.047235
**6333**	242.12608	negative	3.012	$C_{11} H_{18} N_2 O_4$	2.27	0.000832	0.006316
**6447**	352.17318	negative	2.421	$C_{16} H_{24} N_4 O_5$	5.31	0.009043	0.040948
**6740**	356.00096	negative	3.607	$C_7 H_{14} Br N_6 O_4 P$	3.15	0.000396	0.00344
**7057**	648.43521	negative	3.788	$C_{32} H_{56} N_8 O_6$	6.11	0.007121	0.03466

Table S10. Comparison of RCC staging markers in advanced staged RCC (*n* = 41) *vs.* healthy controls (*n* = 176). Fold change (FC) was calculated as the base 2 logarithm of the average intensity ratios between advanced-staged RCC and controls samples. Positive FC values indicate increased abundance in advanced-staged RCC, while negative values indicate higher abundance in control samples. P-values are computed using Welch's *T*-test, and *q*-values represent Benjamini-Hochberg correction. The first 14 metabolomic features has *p* values less than 0.05.

Metabolite/ID	FC	*p*-value	*q*-value	Increase in Advanced RCC (*vs.* Controls)	Increase in Advanced RCC (*vs.* Early RCC)
**4116**	-1.33	1.94E-09	8.40E-08	No	No
**6687**	-1.2	4.14E-09	1.61E-07	No	Yes
**6694**	-2.32	1.35E-08	4.47E-07	No	No
**lys-gly/ gly-lys**	1.87	8.95E-05	1.00E-03	Yes	Yes
**7-aminomethyl-7-carbaguanine**	2.07	9.50E-04	7.01E-03	Yes	Yes
**apo-[3-methylcrotonoyl-CoA:carbon-dioxide ligase (ADP-forming)]**	1.05	1.90E-03	1.22E-02	Yes	Yes
**hydroxyprolyl-asparagine/ asparaginylhydroxyproline**	1.44	1.95E-03	1.25E-02	Yes	Yes
**3163**	1.17	4.33E-03	2.36E-02	Yes	Yes
**5420**	1.95	5.29E-03	2.77E-02	Yes	Yes
**N,N- dimethyl- histidine**	0.87	5.51E-03	2.86E-02	Yes	Yes
**acetone**	0.65	1.03E-02	4.54E-02	Yes	Yes
**pyruvate**	0.4	1.74E-02	6.71E-02	Yes	Yes
**dihydrouridine**	1.07	3.49E-02	1.11E-01	Yes	Yes
**4-guanidinobutanoic acid**	-0.63	3.95E-02	1.22E-01	No	No
**succinic anhydride**	1.03	5.53E-02	1.54E-01	Yes	Yes
**cytosine dimer**	0.29	1.16E-01	2.58E-01	Yes	Yes
**5713**	0.53	1.75E-01	3.35E-01	Yes	Yes
**3-hydroxyanthranilic acid**	0.53	2.32E-01	4.02E-01	Yes	Yes
**diethyl-2-methyl-3-oxosuccinate**	0.69	2.35E-01	4.06E-01	Yes	Yes
**glycine**	0.16	2.58E-01	4.30E-01	Yes	No
**5437**	0.5	2.70E-01	4.42E-01	Yes	Yes
**citrate**	-0.16	3.29E-01	4.89E-01	No	No
**choline**	0.02	8.49E-01	8.98E-01	Yes	Yes
**5045**	-0.02	9.51E-01	9.67E-01	No	Yes
